# A RAC-GEF network critical for early intestinal tumourigenesis

**DOI:** 10.1038/s41467-020-20255-4

**Published:** 2021-01-04

**Authors:** K. A. Pickering, K. Gilroy, J. W. Cassidy, S. K. Fey, A. K. Najumudeen, L. B. Zeiger, D. F. Vincent, D. M. Gay, J. Johansson, R. P. Fordham, B. Miller, W. Clark, A. Hedley, E. B. Unal, C. Kiel, E. McGhee, L. M. Machesky, C. Nixon, A. E. Johnsson, M. Bain, D. Strathdee, S. R. van Hoof, J. P. Medema, K. I. Anderson, S. M. Brachmann, V. M. Stucke, A. Malliri, M. Drysdale, M. Turner, L. Serrano, K. Myant, A. D. Campbell, O. J. Sansom

**Affiliations:** 1 CRUK Beatson Institute, Garscube Estate, Switchback Road, Glasgow, G61 1BD UK; 2 CRUK Cambridge Institute, University of Cambridge, Robinson Way, Cambridge, CB2 ORE UK; 3 Institute of Cancer Sciences, University of Glasgow, Garscube Estate, Switchback Road, Glasgow, G61 1QH UK; 4 EMBL/CRG Systems Biology Research Unit, Centre for Genomic Regulation (CRC), Barcelona, Spain; 5 Universitat Pompeu Fabra (UPF), 08003 Barcelona, Spain; 6 Institute for Theoretical Biology, Humboldt Universität zu Berlin, Berlin, Germany; 7 The Babraham Institute, Babraham Hall, Babraham, Cambridge, CB22 3AT UK; 8 IBAHCM and School of Veterinary Medicine, 464 Bearsden Road, Bearsden, Glasgow, G61 1QH UK; 9 Laboratory for Experimental Oncology and Radiobiology (LEXOR), Center for Experimental Molecular Medicine (CEMM) and Cancer Center Amsterdam, Academic Medical Center, Amsterdam, The Netherlands; 10 Oncode Institute, Academic Medical Center, Amsterdam, The Netherlands; 11 The Francis Crick Institute, Mill Hill Laboratory, London, NW7 1AA UK; 12 Novartis Institutes for BioMedical Research, Klybeckstrasse, 141, 4002 Basel, Switzerland; 13 CRUK Manchester Institute, 553 Wilmslow Road, Manchester, M20 4BX UK; 14 Broad Institute, 415 Main St, Cambridge, MA 02142 United States; 15 Edinburgh Research Centre, The Institute of Genetics and Molecular Medicine, Crewe Road South, Edinburgh, EH4 2XR UK

**Keywords:** Cancer models, Colon cancer

## Abstract

RAC1 activity is critical for intestinal homeostasis, and is required for hyperproliferation driven by loss of the tumour suppressor gene *Apc* in the murine intestine. To avoid the impact of direct targeting upon homeostasis, we reasoned that indirect targeting of RAC1 via RAC-GEFs might be effective. Transcriptional profiling of *Apc* deficient intestinal tissue identified *Vav3* and *Tiam1* as key targets. Deletion of these indicated that while TIAM1 deficiency could suppress *Apc-*driven hyperproliferation, it had no impact upon tumourigenesis, while VAV3 deficiency had no effect. Intriguingly, deletion of either gene resulted in upregulation of *Vav2*, with subsequent targeting of all three (*Vav2*^−/−^ *Vav3*^−/−^ *Tiam1*^−/−^), profoundly suppressing hyperproliferation, tumourigenesis and RAC1 activity, without impacting normal homeostasis. Critically, the observed RAC-GEF dependency was negated by oncogenic KRAS mutation. Together, these data demonstrate that while targeting RAC-GEF molecules may have therapeutic impact at early stages, this benefit may be lost in late stage disease.

## Abstract (web summary)

Loss of small GTPase RAC1 suppresses intestinal tumorigenesis caused by APC loss, but impacts normal intestinal homeostasis. Here, the authors provide an alternative method of reducing RAC1 activity by the combined targeting of three RAC-GEFs and show that this approach delays intestinal tumorigenesis without the detrimental effects on normal intestinal architecture.

## Introduction

RAC1 is a Rho GTPase that exists in two conformational states, an active GTP-bound protein and an inactive GDP-bound protein^1^. Active RAC1 is vital for a multitude of physiological processes, including cytoskeletal re-organisation, cell division and cell migration^2^. It is primarily known for its key role in regulating actin microfilament networks, being a major regulator of lamellipodia formation and cell migration^3,4^, but it is equally important for cell proliferation^5,6^. For example, in non-small cell lung carcinoma, proliferation is dependent upon the transcriptional activation of NF-kB by RAC1^7^, whilst in vitro studies have shown that RAC1 is required for cell proliferation and G2/M progression in a rat fibroblast cell line^6^.

The requirement of RAC1 for transformation was first described in the 1990s^8^, where RAC1 was shown to be overexpressed and its activity increased in many human cancers. In addition, a common activating mutation of RAC1 has recently been discovered in melanoma, and RAC1B, a constitutively active splice-variant of RAC1, is observed in colon cancer^9,10^. Additionally, a recent CRISPR screen carried out by the Sabatini lab has suggested that RAC1 activation may have a critical role in RAS-driven tumourigenesis^11^. We and others have previously shown, using genetically engineered mouse models, that RAC1 is an important downstream effector of APC loss where mutations in the *Apc* gene lead to the initiation of colorectal cancer (CRC)^12–14^. APC is a negative regulator of the WNT signalling pathway and an essential component of the destruction complex that targets β-catenin for degradation by the proteasome^15,16^. Loss of APC results in the translocation and accumulation of β-catenin in the nucleus, activation of the WNT pathway and if targeted to the stem cell compartment, rapid adenoma formation^17,18^. Acute loss of APC in vivo throughout intestinal lineages again drives WNT activation and a crypt progenitor-cell phenotype with increased proliferation and perturbed differentiation leading to a stem cell expansion, mislocalisation of Paneth cells and a reduction in goblet and enteroendocrine lineages^14,17^. Following APC loss, the stem cell markers *Lgr5* and *Olfm4* are upregulated and the number of stem cells present in the intestinal crypt is increased^14^. Loss of RAC1 is sufficient to suppress the hyperproliferation of stem cells and the tumourigenesis caused by APC loss. Mechanistically, this is in part due to a reduction in NF-κB signalling^14^.

RAC-GTPase switches between active and inactive states, and this switching is catalysed by Guanine Nucleotide Exchange Factors (GEFs) and GTPase Activating Proteins (GAPs), respectively^19^. GEFs promote the exchange of GDP for GTP which causes a conformational change that exposes the effector binding region of the GTPase, thus enabling it to transmit signals downstream^20,21^_._ Currently, ~80 mammalian Rho GTPase GEFs have been described^22^, divided into two subfamilies; the Dbl-Pleckstrin Homology domain containing GEFs and DOCK180-related GEFs^23^. Importantly, GEFs are upregulated in numerous human cancers, e.g. PREX1 in melanoma and VAV1 in pancreatic cancer^24,25^.

Given that RAC1 activity is vital to tumour development and progression, there is much focus on RAC1 as a pharmacological target. However, there are multiple difficulties with targeting RAC1 directly, as inhibitors of GTPases are notoriously either inefficient, lacking specificity, or too toxic^26^. Additionally, loss of RAC1 in the mouse is embryonically lethal^27^, and as we have demonstrated, targeted loss of RAC1 from the intestinal epithelium is detrimental to the structure of the intestinal villi. For this reason, a more specific path must be pursued to allow targeting of the cancer-related roles of RAC1 without affecting its physiological roles. One option would be to inhibit specific cancer-associated RAC1-GEFS, an approach exemplified by inhibitors such as NSC23766, a small-molecule inhibitor which blocks the interaction of a subset of GEFs, including TIAM1, with RAC1.

Over recent years, there has been significant interest in the development of a greater understanding of the contribution of the complex network of GEF molecules to cancer development and progression, particularly in the setting of colorectal disease. This has resulted in a wealth of observations related to the contribution of RAC-GEFs such as ASEF1, ASEF2 or TIAM1, both to early adenoma formation, and to pathways critical for control of aggressive cancer cell phenotypes. Intriguing studies have also highlighted GEF independent roles for some of these molecules in colorectal cancer progression^28–30^. In order to better understand the contribution of RAC1-GEFs to phenotypes associated with APC deficiency in colorectal cancer in vivo, we have undertaken comparative transcriptional profiling of APC deficient and control intestinal tissues. Having identified the RAC-GEFs TIAM1, VAV2 and VAV3 as potential candidates, we have gone on to examine their roles in mediating phenotypes associated with APC depletion. Critically, each of these genes has previously been associated with promotion of tumourigenesis. For example, expression of *Vav2* is upregulated in head and neck squamous cell carcinoma^31^, VAV3 is upregulated in Glioblastoma^32,33^, and TIAM1 activity is an important factor in DMBA/TPA driven HRAS mutated papilloma^34,35^. In the context of this study, GEF molecules also have an important function in intestinal tumourigenesis. By way of example, Malliri and colleagues demonstrated that TIAM1 was an important driver of intestinal tumourigenesis in the *Apc*^min/+^ mouse model, in which tumours arise through sporadic loss of heterozygosity of the *Apc* gene. Here, they demonstrated that TIAM1 loss resulted in a suppression of intestinal tumour initiation, though observed that those tumours that did arise were more invasive^30,36^. Moreover, the GEF molecule ARHGEF4 (ASEF1) has been shown to bind to APC in HCT116 and SW480 cell lines, through an interaction which relieves autoinhibition of ARHGEF4 itself, and results in increased activity of CDC42^28,29^, with a further in vivo study into the role of ARHGEF4 and the related molecule ASEF2 in in the *Apc*^min/+^ model, indicating that loss of either resulted in a reduction in both size and number of intestinal adenomas^37^.

We show that although *Rac1* deletion throughout the intestinal epithelium causes defects in intestinal homeostasis, loss of VAV2, VAV3 and TIAM1 is sufficient to strongly suppress cancer phenotypes and tumourigenesis induced by APC loss, whilst leaving the normal intestinal epithelium unperturbed. Additionally, we demonstrate that the role of these GEFs is context specific, as their ability to suppress the APC phenotype is not recapitulated in an in vivo model of hepatocellular tumourigenesis.

Finally, although hyperactivation of the WNT pathway through loss of APC is thought of as the archetypal initiating event in colorectal cancer, and alone is sufficient to drive intestinal polyposis in both human and mouse^38,39^, further oncogenic mutations are required for progression to colorectal cancer^40^. We demonstrate that oncogenic mutation of KRAS, a feature of ~40% of human colorectal cancers^41^, and key driver of resistance to current clinical therapeutic approaches, while remaining sensitive to RAC depletion, drives profound resistance to deletion of VAV2, VAV3 and TIAM1 in the absence of compensatory RAC-GEF expression in vivo.

As a whole, the work presented here clearly demonstrates that targeting RAC-GEFs is a promising avenue for suppression of the RAC1 pathway, and could be highly efficacious in targeting early stage disease. Critically, our work also highlights significant plasticity associated with regulation of RAC1 activity, including compensatory expression of RAC-GEFs, and resistance to RAC-GEF targeting elicited by compounding oncogenic mutation of KRAS. These observations further strongly suggest that targeting RAC1-GEF molecules alone may not be sufficient for cancer therapy in late stage disease.

## Results and discussion

### RAC1 loss perturbs villus homeostasis

**Fig. 1:**
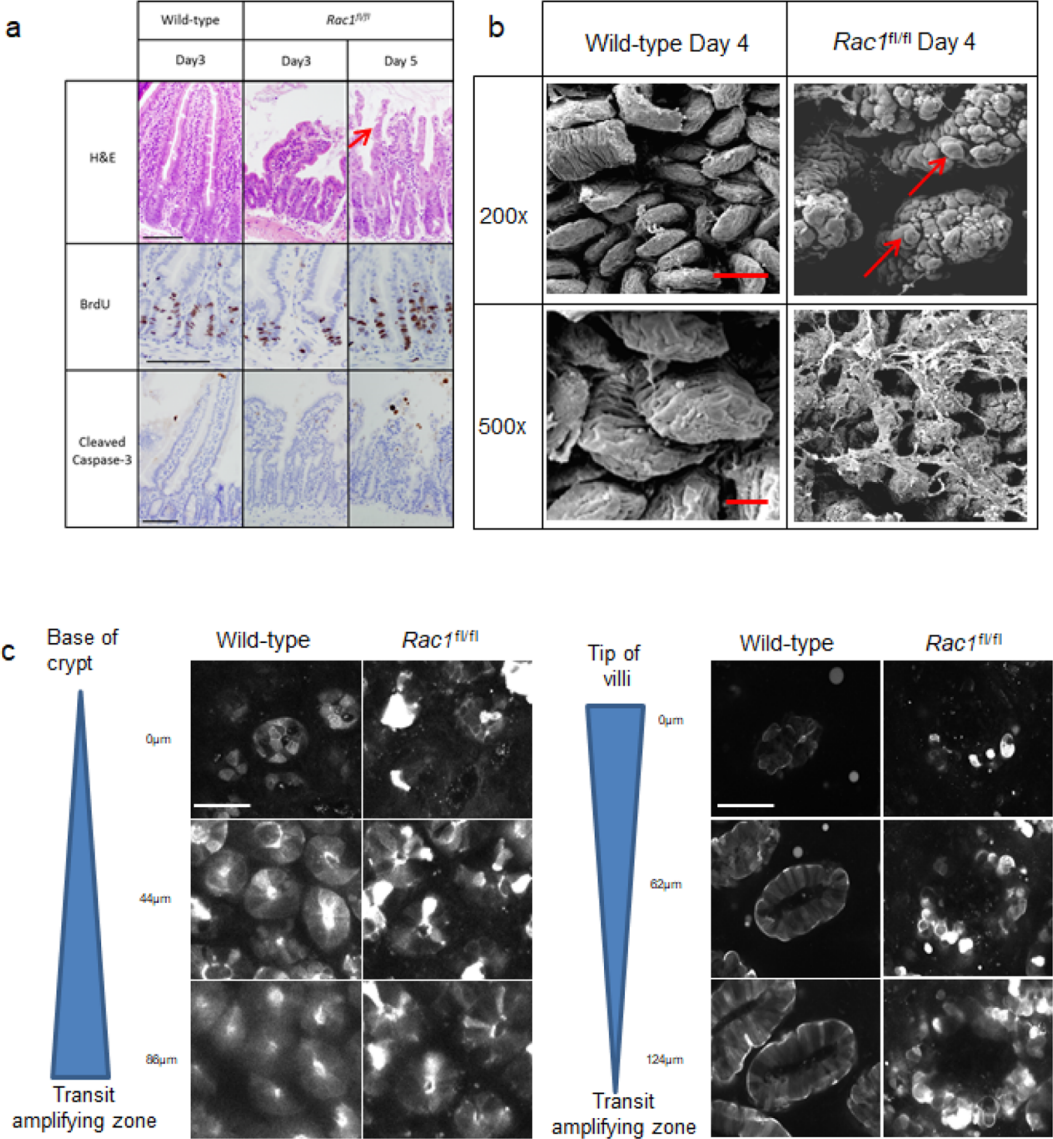
Loss of Rac1 perturbs villus homeostasis. **a** Images showing H&E, BrdU incorporation and Cleaved caspase-3 IHC in *Vil-CreER*^*T2*^ (wild-type), *Vil-CreER*^*T2*^ *Rac1*^*fl/fl*^ (*Rac1*^*fl/fl*^*)* mice day 3 post induction and *Vil-CreER*^T2^ *Rac1*^*fl/fl*^ (*Rac1*^*fl/fl*^*)* mice 3 and 5 days post induction. Red arrow indicates disintegrating villus. Scale bar represents 100 μm in each case (See amplified images in Supplementary Fig. 1d–f). **b** Scanning EM on *Vil-CreER*^*T2*^ (Wild-type) or *Vil-CreER*^*T2*^ *Rac1*^*fl/fl*^ (*Rac1*^*fl/fl*^) intestines 4 days post induction. Red arrows indicate rounded, blebbing cells. Scale bar represents 100 μm in the upper panels and 50 μm in the lower panels. **c** *Vil-CreER*^*T2*^ driving expression of *LifeAct*-GFP in wild-type and *Rac1*^*fl/fl*^ intestines 4 days post induction. Scale bar represents 50 μm.

We have previously shown that *Rac1* deletion suppresses the “crypt progenitor-like” phenotype, in addition to the tumourigenesis resulting from APC deficiency^14^. This study suggested that targeting of RAC1 in intestinal cancer could be beneficial. Given that therapeutic targeting of RAC1 in CRC would require suppression of activity over a prolonged period of time, we sought to address the consequences of RAC1 loss throughout the intestine, mimicking the action of an inhibitor. We therefore generated *Vil-CreER*^*T2*^ *Rac1*^*fl/fl*^ mice and treated them with 2 mg tamoxifen for 2 days to delete *Rac1* throughout the intestinal epithelium and observed the impact over time^42,43^. At 5 days post induction, mice developed symptoms of intestinal ill-health (weight loss and hunching), were euthanised and the intestines harvested. Histological analyses show that while crypt structure, including epithelial proliferation, appeared relatively unperturbed, there was a marked change in villus architecture (Fig. 1a), with villi dramatically shortened and exhibiting marked disintegration (Fig. 1a, arrow in Fig. 1a indicates disintegration, Supplementary Fig. 1B, D, E with Supplementary Fig. 1A indicating the regions of interest of the crypt-villus) and apoptosis (Fig. 1a and Supplementary Fig. 1C, F). To investigate this more closely, we performed scanning EM of the intestinal epithelium and found an altered morphology of the villus, with clear bleb-like structures (as indicated by arrow Fig. 1b). Given the role of RAC1 in maintaining the actin cytoskeleton, we reasoned that the collapse of the villus structure and premature cell sloughing may be due to a perturbed actin network. To examine this, we crossed the *Vil-CreER*^*T2*^ *Rac1*^*fl/fl*^ mice to mice carrying the LifeAct-GFP reporter^42,43^. Gross perturbations in F-actin structures were observed in the villus but not in the crypts of these mice, as shown by intravital fluorescence imaging (Fig. 1c). Together our data show that a reduction in RAC1 in the intestinal epithelium results in detrimental effects on epithelial integrity and normal barrier function.

To verify the efficiency of deletion of *Rac1* from this mouse model, and show specificity of the *Vil-CreER*^*T2*^ promoter, we designed a *Rac1* BaseScope probe which allowed detection of exons 4 and 5 (the specific exons lost upon induction). When APC is lost, *Rac1* is expressed throughout the intestine in both the epithelial and stromal compartments. However, following targeted deletion of *Rac1* alongside APC deletion using the *Vil-CreER*^*T2*^ promoter, we observed a striking loss of Rac1 expression specifically in the intestinal epithelium (Supplementary Fig. 2A). This is further verified by reduction in RAC1 protein as shown by immunohistochemical analysis using a RAC1 antibody (phospho S71; Supplementary Fig. 2B). Interestingly, despite the similarities in signalling pathways between RAC1 and CDC42 and being close homologues^4,5,44^, CDC42 expression is unaffected by loss of RAC1 (Supplementary Fig. 2C).

### VAV3 and TIAM1 are upregulated following APC loss

**Fig. 2:**
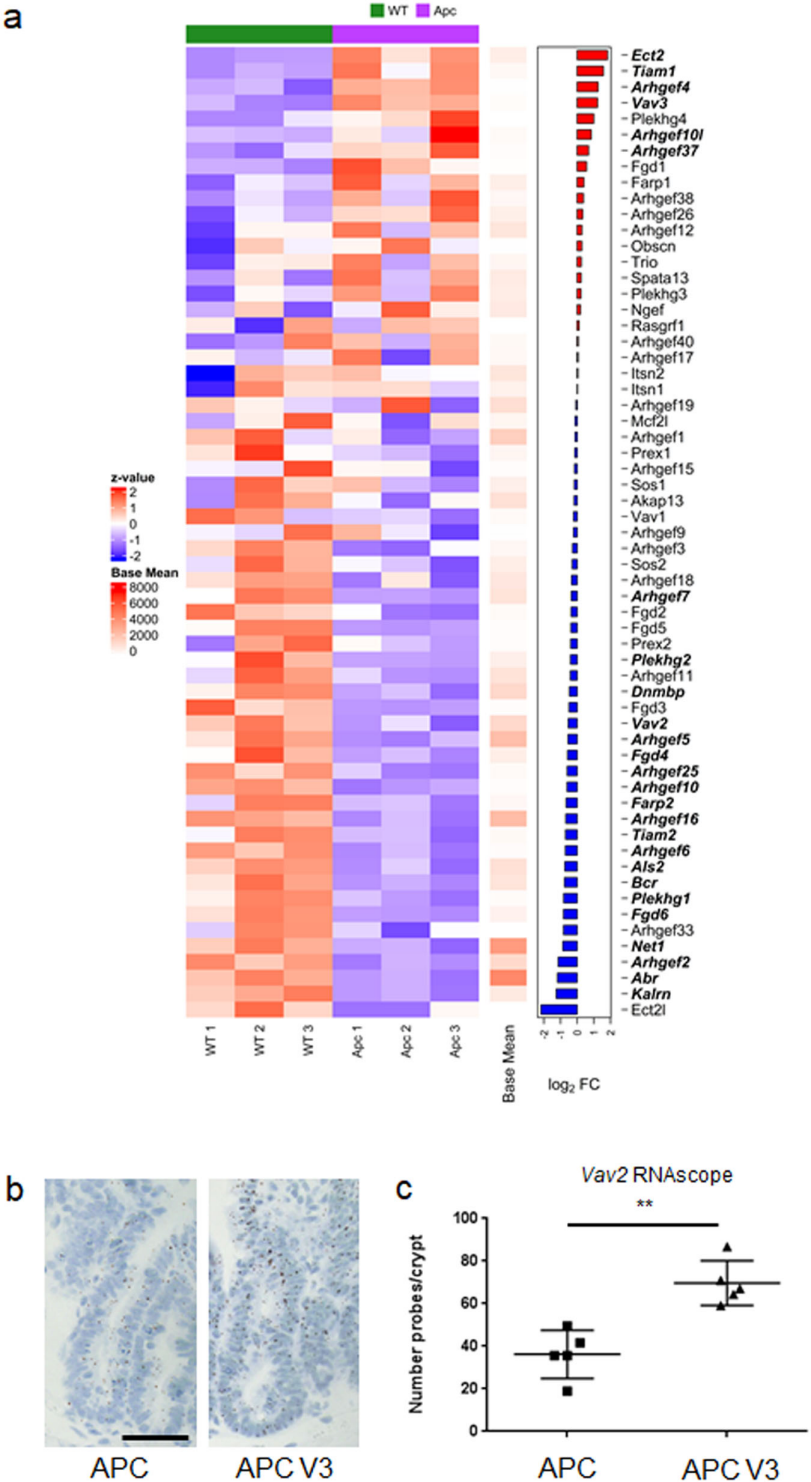
VAV3 and TIAM1 are upregulated following APC loss. **a** Heatmap derived from RNA-seq analysis comparing whole tissue from wild-type (*Vil-CreER*^*T2*^) and APC intestines (*Vil-CreER*^*T2*^ *Apc*^*fl/fl*^) *n* = 3 biologically independent animals for both APC and WT intestinal tissue. Log_2_FC of GEF expression displayed on the right, genes significantly deregulated displayed in bold (FDR < 0.05) displayed in bold. **b** RNAscope of *Vav2* in the intestinal epithelium of *Vil-CreER*^*T2*^ *Apc*^*fl/fl*^ (APC) and *Vil-CreER*^T2^ *Apc*^*fl/fl*^, *Vav3*^−/−^ (APC V3) mice, Scale bar represents 100 μm. **c** Quantification of *Vav2* RNAscope from *Vil-CreER*^T2^ *Apc*^*fl/fl*^ and *Vil-CreER*^T2^ *Apc*^*fl/f*l^ *Vav3*^−/−^ intestines. *N* = 5 biologically independent animals for each genotype. *P* = 0.0079 as determined by a two-tailed Mann–Whitney test. Data are presented as mean values ±SD.

In light of the intestinal toxicity associated with RAC1 loss, we used RNA-seq analysis to profile the GEFs expressed in the murine small intestine, comparing wild-type tissue to APC-deficient tissue and observed distinct differential expression as shown by a principle component analysis (PCA; Supplementary Fig. 3A). This analysis showed that *Ect2, Tiam1, Arhgef4* and *Vav3* were highly upregulated following APC loss (Fig. 2a and Supplementary Fig. 3A). Although *Ect2* was the most induced GEF following APC loss and has previously been associated with tumourigenesis in the brain^32^, it is also known that constitutive knockout of *Ect2* in the mouse is embryonic lethal^45^. *Ect2* has also been identified as a common essential gene through CRISPR and RNAi screening (DEPMAP project^46^) raising concerns related to toxicity that may be encountered through therapeutic targeting of ECT2. Additionally, ARHGEF4 has been identified as a CDC42 specific GEF^28^. As such, our research initially focussed upon targeting of the induced GEFs TIAM1 and VAV3 in intestinal tumourigenesis driven by APC loss. Intriguingly, VAV1 is sparsely expressed and predominantly seen in stromal tissue, despite showing high sequence similarity to VAV2 and VAV3 (Supplementary Fig. 3B–E). Interestingly, in wild-type intestine, VAV2 is predominantly expressed in the stromal compartment, while following loss of APC, it becomes highly expressed in the intestinal epithelium (Supplementary 4A, B). This indicates that while expression of VAV2 may not appear to be induced in whole intestinal tissue, it is induced in the intestinal epithelium following APC depletion and as such is of significant interest. To investigate potential compensation between VAV2 and VAV3, we examined *Vav2* expression in *Vil-CreER*^*T2*^ *Apc*^*fl/fl*^ *Vav3*^–/–^ mice by RNAscope and observed a significant increase in *Vav2* expression compared to *Vil-CreER*^*T2*^ *Apc*^*fl/fl*^ mice (an average of 36.27 probes per crypt were observed in *Vil-CreER*^*T2*^ *Apc*^*fl/fl*^ compared to 69.64 probes per crypt, a fold change of 1.92; Fig. 2b, c).

The observed induction of VAV2 and VAV3 in the murine intestinal epithelium following *Apc* deletion is also reflected in human disease. Recent efforts to classify the molecular subtypes of human colorectal cancer (CRC) based upon large scale genomic, transcriptional and proteomic data have identified four major consensus molecular subtypes (CMS1-4)^47^. Of these, CMS2 is most closely associated with APC loss and altered regulation of the canonical WNT pathway, and as such most closely related to our in vivo model. Interrogation of the transcriptional profiles of *VAV2, VAV3* and *TIAM1* in a patient-derived CRC dataset indicated that expression of both *VAV2* and *VAV3* is particularly enriched in the APC loss associated CMS2 subtype. TIAM1 expression is found in all CMS subtypes of CRC, however it is markedly lower than that of VAV2 and VAV3 (Supplementary Fig. 5).

### Loss of one or two intestinal GEFs is insufficient to rescue the phenotype caused by APC loss

Based on the GEF expression levels and the observed compensation between VAV2 and VAV3, we examined the impact of *Tiam1* deletion and of combined *Vav2/Vav3* knockout on the phenotypes caused by intestinal APC loss.

**Fig. 3:**
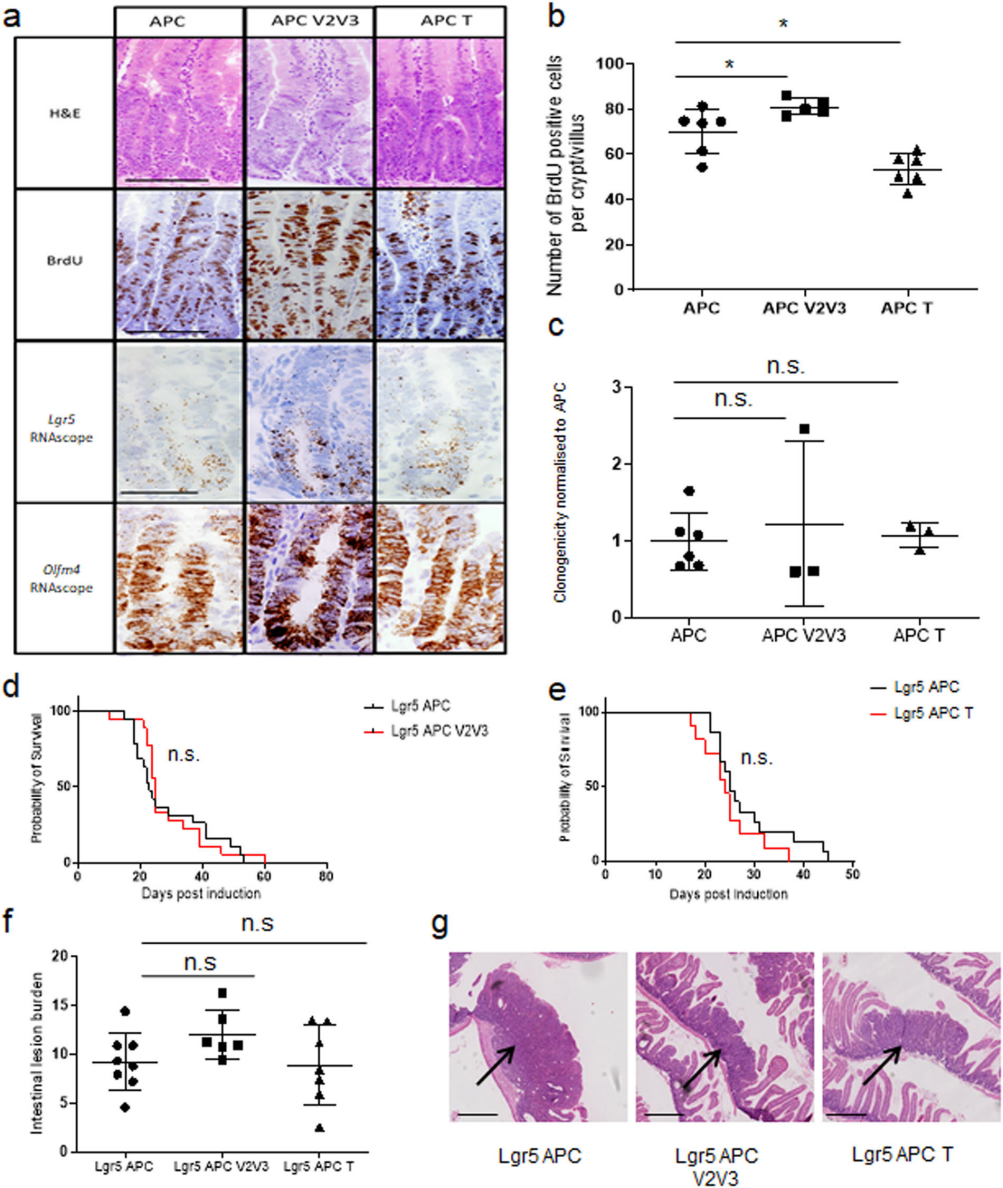
Loss of a single or double GEF is unable to rescue the *Apc*^*fl/fl*^ phenotype. **a** H&E, BrdU incorporation and RNAscope for *Lgr5* and *Olfm4* on *Vil-CreER*^T2^ *Apc*^*fl/fl*^ (APC), *Vil-CreER*^T2^ *Apc*^*fl/fl*^, *Vav2*^−/−^, *Vav3*^−/−^ (APC V2V3) and *Vil-CreER*^T2^ *Apc*^*fl/fl*^ *Tiam1*^−/−^ (APC T). Scale bar represents 100 μm for H&E and BrdU and 50 μm for RNAscope images. **b** Quantification of BrdU positive cells. *N* = 6, 5 and 6 biologically independent animals for *Vil-CreER*^T2^ *Apc*^*fl/fl*^ (APC), *Vil-CreER*^T2^ *Apc*^*fl/fl*^, *Vav2*^−/−^, *Vav3*^−/−^ (APC V2V3; *p* = 0. 0303 as determined by a two-tailed Mann–Whitney) and *Vil-CreER*^T2^ *Apc*^*fl/fl*^ *Tiam1*^−/−^ (APC T; *p = 0.0260, as determined by a two-tailed Mann–Whitney) respectively. Data are presented as mean values ±SD. **c** Quantification of clonogenicity assay of intestinal organoids. *N* = 6, 3 and 3 biologically independent animals for *Vil-CreER*^T2^ *Apc*^*fl/fl*^ (APC), *Vil-CreER*^T2^ *Apc*^*fl/fl*^, *Vav2*^−/−^, *Vav3*^−/−^ (APC V2V3; *p* = 0.5476, as determined by a two-tailed Mann–Whitney) and *Vil-CreER*^T2^ *Apc*^*fl/fl*^ *Tiam1*^−/−^ (APC T) respectively (*p* = 0.5476, as determined by a two-tailed Mann–Whitney). Data are presented as mean values ±SD. **d**, **e** Survival of *Lgr5-EGFP-IRES-creER*^*T2*^ *Apc*^*fl/fl*^ (Lgr5 APC), *Lgr5-EGFP-IRES-creER*^*T2*^ *Apc*^*fl/fl*^ *Vav2*^−/−^, *Vav3*^−/−^ (Lgr5 APC V2V3) and *Lgr5-EGFP-IRES-creER*^*T2*^ *Apc*^*fl/fl*^ *Tiam1*^−/−^ (Lgr5 APC T). **d** *N* = 19 and 18 biologically independent animals for Lgr5 APC and Lgr5 APC V2V3 respectively (*p* = 0.7142, as determined by Log-rank (Mantel-Cox) test). **e** *N* = 15 and 10 biologically independent animals for Lgr5 APC and Lgr5 APC T respectively (under the control of *Lgr5-EGFP-IRES-creER*^*T2*^). (*p* = 0.1982, as determined by Log-rank (Two-tailed Mantel-Cox test). The same Lgr5 APC control cohort was used in both **d** and **e** as well as in Fig. 4f and Supplementary Fig. 6D. **f** Quantification of intestinal tumour burden following APC loss in the Lgr5 stem cell compartment. Tumour burden is determined as percentage of intestine which is covered by lesion or adenomas. *Lgr5-EGFP-IRES-creER*^*T2*^ *Apc*^*fl/fl*^ (APC; *n* = 9 biologically independent animals) vs *Lgr5-EGFP-IRES-creER*^*T2*^ *Apc*^*fl/fl*^ *Vav2*^−/−^, *Vav3*^−/−^ (APC V2V3; *n* = 6 biologically independent animals) *p* = >0.9999 as determined by a two-tailed Kruskal–Wallis with Dunn’s multiple comparisons test. *Lgr5-EGFP-IRES-creER*^*T2*^ *Apc*^*fl/fl*^ (APC) vs *Lgr5-EGFP-IRES-creER*^*T2*^ *Apc*^*fl/fl*^ *Tiam1*^−/−^ (APC T; *n* = 7 biologically independent animals), *p* = 0.7532 as determined by a two-tailed Kruskal–Wallis with Dunn’s multiple comparisons test. Data are presented as mean values ±SD. **g** Solid adenomas (H&E) were observed in each genotype, indicated by the arrow. Scale bar represents 500 μm.

We generated *Vil-CreER*^*T2*^ *Apc*^*fl/fl*^ *Vav2*^−/−^ *Vav3*^−/−^ (APC V2V3) and *Vil-CreER*^*T2*^ *Apc*^*fl/fl*^ *Tiam1*^−/−^ (APC T) mice and compared these to control *Vil-CreER*^*T2*^ *Apc*^*fl/fl*^ (APC). We induced APC loss in the intestine by Cre-induction with tamoxifen, and analysed the mice 4 days later. At this time, the APC-deficient intestines developed the expected “crypt progenitor-like” phenotype, with mice developing large hyperproliferative crypts, alongside an increase in stem cell markers (visualised here by RNAscope for *Lgr5* and *Olfm4*; Fig. 3a)^17^. To validate loss of the GEFs, immunohistochemical analysis was performed (Supplementary Fig. 4B). Here we demonstrate a loss of VAV2 and VAV3 expression in APC V2V3 intestines. The expression of VAV2 was unaffected by loss of TIAM1 (when compared to APC). Additionally, using CTGF as a marker for TIAM1 activity^48^, we observed an increase in cytoplasmic CTGF upon loss of TIAM1 in APC T, indicating a loss of TIAM1 activity.

Loss of VAV2 and VAV3 slightly exacerbated the crypt progenitor phenotype driven by APC deficiency, resulting in a slight but significant increase in crypt cell proliferation (Fig. 3a, b and Supplementary Fig. 6A–C). In contrast, *Tiam1* knockout reduced hyperproliferation associated with APC loss, but did not impact the increase in stem cell marker expression (Fig. 3a, b and Supplementary Fig. 6A–C). To examine the stem cell phenotype functionally, we used an in vitro clonogenicity assay where APC-deficient intestinal single-cell suspensions are able to re-grow into organoid spheres within 5 days. Consistent with the finding that deficiency of both VAV2 and VAV3 or TIAM1 had no effect on stem cell marker expression dependent upon APC loss, intestinal cells from mice lacking both VAV2 and VAV3 or TIAM1 retained the ability to form organoids (Fig. 3c).

Finally, to investigate whether GEF deficiency had a functional effect on tumourigenesis, we generated *Lgr5-EGFP-IRES-creER*^*T2*^ *Apc*^*fl/fl*^ (Lgr5 APC), *Lgr5-EGFP-IRES-creER*^*T2*^ *Apc*^*fl/fl*^ *Vav2*^−/−^ *Vav3*^−/−^ (Lgr5 APC V2V3) and *Lgr5-EGFP-IRES-creER*^*T2*^ *Apc*^*fl/fl*^ *Tiam1*^−/−^(Lrg5 APC T) mice, examining the role of these GEFs when *Apc* is deleted solely from the intestinal stem cells. Homozygous deletion of *Apc* was induced in these mice using a 4-day protocol of tamoxifen treatment^14^, with mice aged until they developed signs of intestinal neoplasia (weight loss and hunching). This is a rapid model of tumourigenesis, in contrast to other models where initiation of tumourigenesis is dependent upon the spontaneous loss of the second copy of *Apc*. The majority of the Lgr5 APC mice reach endpoint and display clinical signs by day 25 post induction (Fig. 3d, e)^18^. Neither VAV2/VAV3-deficiency nor TIAM1-deficiency slowed tumourigenesis caused by APC loss (Fig. 3d, e). While these ageing cohorts were carried out independently, each cohort had its own control Lgr5 APC arm between which no survival difference was observed (Supplementary Fig. 6D). Histologically, the adenomas that formed in Lgr5 APC V2V3 mice or in Lgr5 APC T mice were similar to those in mice lacking only APC in lesion burden, lesion structure and cellular proliferation at endpoint (Fig. 3f, g and Supplementary Fig. 6C).

Taken together, these data show that the single deletion of the RAC-GEF *Tiam1* or the double deletion of *Vav2* and *Vav3* was insufficient to recapitulate the phenotype caused by RAC1 deficiency (namely an extension in survival following APC loss)^14^. Moreover, TIAM1 deficiency, which resulted in reduced proliferative capacity following acute deletion of APC (Fig. 3b), was unable to extend survival in the Lgr5 APC ageing model of CRC, suggesting that reduced proliferation alone is insufficient to impair efficient tumourigenesis. It should be noted that our data differ somewhat from the previous study in the *Apc*^min/+^ mouse, which showed that TIAM1 loss slows tumourigenesis^30^. We believe this is due to differences between the two models. The *Apc*^min/+^ model of tumourigenesis is dependent on a spontaneous loss of the second copy of *Apc*, whereas the *Lgr5-EGFP-IRES-creER*^*T2*^ *Apc*^*fl/fl*^ (Lgr5 APC) model has both copies of *Apc* lost at induction which results in rapid tumourigenesis elicited in the LGR5 positive stem cell compartment. It is possible that the effect of TIAM1 on the hyperproliferative phenotype suppresses growth early on, with some APC-deficient clones potentially arising outside of the stem cell zone.

### Loss of VAV2, VAV3 and TIAM1 are required to suppress the cancer phenotype caused by APC loss

**Fig. 4:**
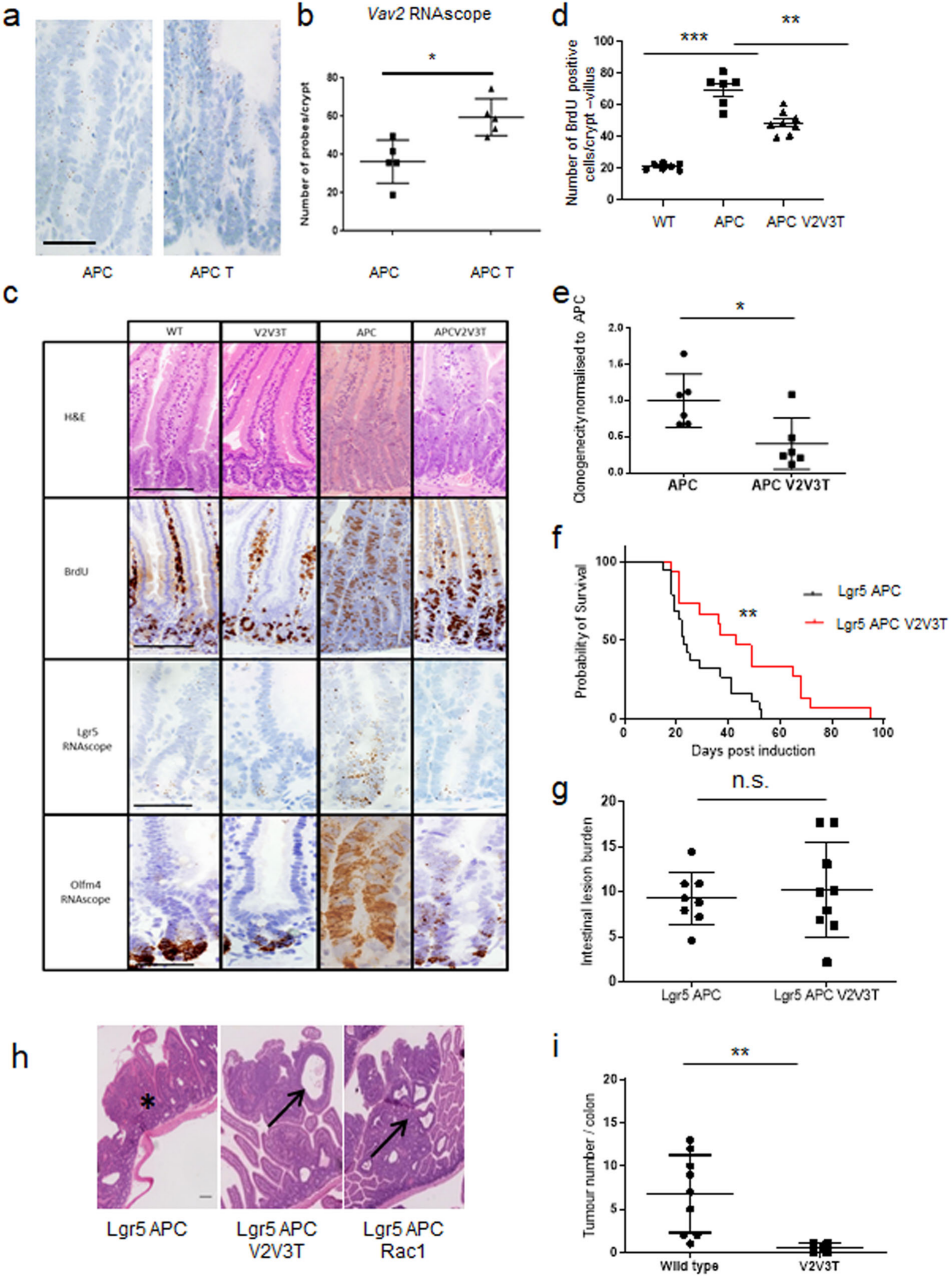
Loss of three GEFs is able to suppress the loss of *Apc* phenotype. **a** RNAscope staining for *Vav*2 in intestine from *Vil-CreER*^T2^ *Apc*^*fl/fl*^ (APC) and *Vil-CreER*^T2^ *Apc*^*fl/fl*^ *Tiam1*^−/−^ (APC T) intestines. Scale bar represents 100 μm. **b** Quantification of *Vav2* RNAscope in *Vil-CreER*^T2^ *Apc*^*fl/fl*^ (APC) and *Vil-CreER*^T2^ *Apc*^*fl/fl*^ *Tiam1*^−/−^ (APC T) intestines. *N* = 5 biologically independent animals for each genotype *p = 0.0159 as determined by a two-tailed Mann–Whitney test. Data are presented as mean values ±SD. **c** Images for H&E and BrdU incorporation and RNAscope for *Lgr5* and *Olfm4* on wild-type (WT), *Vav2*^−/−^, *Vav3*^*−/*^ *Tiam1*^−/−^ (V2V3T), *Vil-CreER*^T2^ *Apc*^*fl/fl*^ (APC) and *Vil-CreER*^T2^ *Apc*^*fl/fl*^, *Vav2*^−/−^ and *Vav3*^−/−^*Tiam1*^−/−^ (APC V2V3T). Scale bar represents 100 μm for H&E and BrdU and 50 μm for RNAscope images. **d** Quantification of BrdU positive cells. *N* = 7, 6 and 8 biologically independent animals for wild-type, *Vil-CreER*^T2^ *Apc*^*fl/fl*^ (APC) and *Vil-CreER*^T2^ *Apc*^*fl/fl*^, *Vav2*^−/−^ and *Vav3*^−/−^ *Tiam1*^−/−^ (APC V2V3T) respectively. WT vs APC ***p = 0.0006, APC vs APC V2V3T *p = 0.0013 as determined by a one-tailed Mann–Whitney test. Control data (APC) as in Fig. 3c. Data are presented as mean values ±SD. **e** Quantification of clonogenicity assay of intestinal organoids. *N* = 6 biologically independent cell lines for both *Vil-CreER*^T2^ *Apc*^*fl/fl*^ (APC) and *Vil-CreER*^T2^ *Apc*^*fl/fl*^, *Vav2*^−/−^, *Vav3*^−/−^ *Tiam1*^−/−^ (APC V2V3T). *p = 0.0260 as determined by a two-tailed Mann–Whitney test. **f** Compared to *Lgr5-EGFP-IRES-creER*^*T2*^ *Apc*^*fl/fl*^ (Lgr5 APC), *Lgr5-EGFP-IRES-creER*^*T2*^ *Apc*^*fl/fl*^ *Vav2*^−/−^, *Vav3*^−/−^ *Tiam1*^−/−^ (Lgr5 APC V2V3T) mice have a significant survival advantage. *N* = 19 and 15 biologically independent animals for Lgr5 APC and Lgr5 APC V2V3T respectively. **p = 0.0091 as determined by a two-tailed Log-rank (Mantel-Cox) test. Lgr5 APC control cohort is the same cohort as used in Fig. 3d, e as well as Supplementary Fig. 6D. Data are presented as mean values ±SD. **g** Quantification of intestinal tumour burden at clinical endpoint following APC loss in the Lgr5 stem cell compartment. *N* = 8 and 9 biologically independent animals for *Lgr5-EGFP-IRES-creER*^*T2*^ *Apc*^*fl/fl*^ (Lgr5 APC) and *Lgr5-EGFP-IRES-creER*^*T2*^ *Apc*^*fl/fl*^ *Vav2*^−/−^, *Vav3*^−/−^ *Tiam1*^−/−^ (Lgr5 APC V2V3T) respectively. *p* = 0.9522 as determined by a two-tailed Mann–Whitney test. Data are presented as mean values ±SD. **h** Solid adenomas (asterisk) were observed in the *Lgr5-EGFP-IRES-creER*^*T2*^ *Apc*^*fl/fl*^ (Lgr5 APC) cohort, whereas cystic adenomas were observed (H&E) in *Lgr5-EGFP-IRES-creER*^*T2*^ *Apc*^*fl/fl*^ *Vav2*^−/−^, *Vav3*^−/−^ *Tiam1*^−/−^ (Lgr5 APC V2V3T) and *Lgr5-EGFP-IRES-creER*^*T2*^ *Apc*^*fl/fl*^*, Rac1*^*fl/fl*^ (Lgr5 APC Rac1), as indicated by arrows. Scale bar represents 100 μm. **i** Intestinal tumour number in an AOM-DSS colitis-associated model of tumourigenesis. Study was carried out on wild-type mice or *Vav2*^−/−^, *Vav3*^−/−^ *Tiam1*^−/−^ mice. *N* = 9 and 4 biologically independent animals for wild-type or *Vav2*^−/−^, *Vav3*^−/−^ *Tiam1*^−/−^ respectively. **p = 0.0042 as determined by a one-tailed Mann–Whitney Test. Data are presented as mean values ±SD.

Given the increased *Vav2* expression in *Vil-CreER*^*T2*^ *Apc*^*fl/fl*^ *Vav3*^–/–^ mice, we also assayed *Vav2* expression in *Vil-CreER*^*T2*^ *Apc*^*fl/fl*^ *Tiam1*^−/−^ intestines and observed a significant increase by RNAscope analysis (an average of 36.27 probes per crypt were observed in *Vil-CreER*^*T2*^ *Apc*^*fl/fl*^ mice compared to 59.45 probes per crypt in *Vil-CreER*^*T2*^ *Apc*^*fl/fl*^ *Tiam1*^−/−^ mice, a fold change of 1.64; Fig. 4a, b). Additionally, we performed IHC analysis of VAV2 on these samples and observed strong epithelial staining in *Vil-CreER*^*T2*^ *Apc*^*fl/fl*^*, Tiam*^−/−^ mice (Supplementary Fig. 4B). Given the upregulation of transcription of *Vav2* and the high expression following loss of TIAM1 alongside APC, we therefore reasoned that VAV2 might also compensate for TIAM1 and thus generated *Vav2*^−/−^ *Vav3*^−/−^ *Tiam1*^−/−^ triple-GEF whole body knockout mice (V2V3T). These mice are viable and exhibit normal intestinal homeostasis (Fig. 4c). We intercrossed triple-GEF knock-out mice with *Vil-CreER*^*T2*^ *Apc*^*fl/fl*^ mice to generate *Vil-CreER*^*T2*^ *Apc*^*fl/fl*^ *Vav2*^−/−^ *Vav3*^−/−^ *Tiam1*^−/−^ (APC V2V3T), in which APC loss could be induced by tamoxifen. Loss of VAV2, VAV3 and TIAM1 expression in this system was demonstrated by immunohistochemical staining (Supplementary Fig. 4B). This model was then used to investigate whether triple-GEF deficiency was sufficient to modify the crypt progenitor-like phenotype caused by APC loss. Four days after induction of APC loss, triple-GEF deficiency resulted in a significant decrease in intestinal cell proliferation (as shown by BrdU incorporation) compared to controls, to a similar level caused by TIAM1 deficiency alone (Figs. 3b and 4c, d). In contrast, the triple-GEF deficient mice (V2V3T) also showed a marked reduction in the expression of the stem cell marker *Lgr5*, whilst *Olfm4* remained largely unchanged (Fig. 4c and Supplementary Fig. 7). We validated this observation through the clonogenicity assay, where cells lacking the three GEFs, as well as APC loss (APC V2V3T), exhibited reduced spheroid-forming capacity compared to APC loss alone or to our previous experiments where 1 or 2 GEFs were lacking (Fig. 4e). Given that we observed not only a reduction in cell proliferation but also a triple-GEF dependent reduction in stem cell marker expression, we assessed whether deletion of these three GEFs was also sufficient to modify tumourigenesis caused by APC loss.

We generated *Lgr5-EGFP-IRES-creER*^*T2*^ *Apc*^*fl/fl*^ *Vav2*^−/−^ *Vav3*^−/−^ *Tiam1*^−/−^ (Lgr5 APC V2V3T) mice to directly compare with *Lgr5-EGFP-IRES-creER*^*T2*^ *Apc*^*fl/fl*^ mice (Lgr5 APC) to determine the effect of triple-GEF deficiency on tumourigenesis. *Apc* deletion was induced as described above, and was performed within the same breeding colony as the Lgr5 APC V2V3 cohort; as such the Lgr5 APC survival data are the same for both ageing studies. Importantly, mice lacking all 3 GEFS (Lgr5 APC V2V3T) showed a marked delay in tumourigenesis when compared to control Lgr5 APC mice (*p* = 0.0091 as determined by Mantel–Cox log rank, Median times to intestinal tumourigenesis 43 days vs 23 days; Fig. 4f). As previously, tumour burden at endpoint was consistent between these two cohorts (Fig. 4g). In addition to enhanced survival, histological differences were observed; while adenomas which developed in control animals were solid (indicated by asterisk in Fig. 4h), those that developed in Lgr5 APC V2V3T mice were more cystic (Indicated by arrow in Fig. 4h). This was reminiscent of the adenomas observed in *Lgr5-EGFP-IRES-creER*^*T2*^ *Apc*^*fl/fl*^ *Rac1*^*fl/fl*^ mice (Fig. 4h)^14^, suggesting that loss of VAV2, VAV3 and TIAM1 could phenocopy the effect of RAC1 loss. To confirm the ability of these three GEFS to suppress colorectal carcinogenesis, we performed an Azoxymethane/Dextran Sodium Sulfate (AOM/DSS) colitis-associated cancer protocol and found that the triple-GEF knockout mice (V2V3T) developed far fewer tumours (Fig. 4i).

Taken together, our data show that these three GEFs, VAV2, VAV3 and TIAM1 are required for the crypt progenitor phenotype and tumourigenesis caused by APC loss in the intestinal stem cell compartment. Deficiency in these three GEFs thus faithfully recapitulates the *Rac1*-deletion phenotype. It is interesting to note that tumourigenesis was only affected when the stem cell marker levels were reduced and clonogenicity in culture impaired, reinforcing the importance of stemness for tumourigenesis. It should also be noted that in the presence of functional APC, triple-GEF knockout intestines had similar levels of intestinal stem cell markers compared to wild-type mice; thus the reduced tumourigenesis was not simply a reflection of fewer intestinal stem cells (Fig. 4c and Supplementary Fig. 7C).

Given that the stem cell targets are known to be driven by WNT signalling, we next examined expression of a broader set of WNT targets and nuclear localisation of β-catenin in the triple-GEF mice following APC loss. In APC-deficient mice, we saw a marked induction in the levels of nuclear β-catenin and induction of targets such as CD44, SOX9, *c-Myc* and *Axin2*. Consistent with our previous studies where we deleted *Rac1*, loss of the three GEFS did not reduce the accumulation of β-catenin in the nucleus^14^ or the expression of CD44, SOX9 or *c-Myc*. In contrast, there was a marked downregulation in the levels of *Axin2*, a well-established component of the destruction complex^49^, again supporting the critical role of RAC1 activity downstream of APC loss (Supplementary Figs. 8 and 9). These data specifically address the potential of a therapeutic window for targeting RAC1. Whilst we have previously shown that loss of RAC1 has a catastrophic effect on the gut (Fig. 1a), targeting of specific RAC-GEFs is capable of suppressing proliferation whilst not impacting the structural integrity of the intestine.

These collective observations are consistent with previous work which has shown that whilst RAC1 is not required for the nuclear accumulation of β-catenin, it is required to mediate phosphorylation of β-catenin at residues S191 and S605 by JNK2, resulting in enhanced activation of WNT-dependent genes^50^. In the absence of these WNT targets, the transforming programme of APC loss, which drives the crypt progenitor phenotype cannot be fully established, with key functional targets such as Axin2 being reduced. This provides a ready explanation for the delay in tumourigenesis in these triple-GEF knockout mice.

### The role of VAV2, VAV3 and TIAM1 on WNT-driven processes is intestine-specific

One important question that remained was how specific this transcriptional network of GEF expression resulting from WNT activation was to intestinal disease. As mentioned previously, triple knockouts are viable and fertile with normal intestinal homeostasis, suggesting little impact on WNT signalling under homeostatic conditions. For this reason, we examined potential induction and functional relevance of these RAC-GEFS in models of hepatocellular disease, a second, independent in vivo model of WNT-driven hyperproliferation. It has previously been shown that loss of APC in the adult murine liver results in an increase in nuclear β-catenin accumulation and *c-Myc* expression, which is associated with hyperproliferation and hepatomegaly^51,52^. We induced loss of APC in the liver using AAV-TBG-Cre^53^. Consistent with previous studies, we observed nuclear accumulation of β-catenin and an increase in liver size in this setting (Supplementary Fig. 10A, B). Interestingly, when we carried out the deletion of *Apc* alongside deletion of *Rac1* using AAV-TBG-Cre, or in the context of mice lacking VAV2, VAV3 and TIAM1, we did not observe rescue of WNT-driven phenotypes; more specifically we saw no impact upon liver weight or liver enzyme production (Supplementary Fig. 10B–E). While this experiment is not able to demonstrate a critical role for VAV2, VAV3 or TIAM1 for RAC1 activation in hepatocellular proliferation, it does critically demonstrate that the requirement for RAC1 activity in WNT-driven hyperproliferation is not uniform. This further indicates that targeting of RAC1 regulatory molecules for anti-cancer impact may prove to be highly context dependent.

### Intestinal RAC activity is reduced in triple-GEF knockout mice

In order to examine the effect of VAV2, VAV3 and TIAM1 deficiency on RAC1 activity in the intestine, we initially looked at the levels of total RAC1. Immunohistochemical analysis showed loss of the GEFs alongside APC resulted in no discernible difference in the amount of total RAC1 present when compared to *Vil-CreER*^*T2*^ *Apc*^*fl/fl*^ mice alone (Supplementary Fig. 11).

**Fig. 5:**
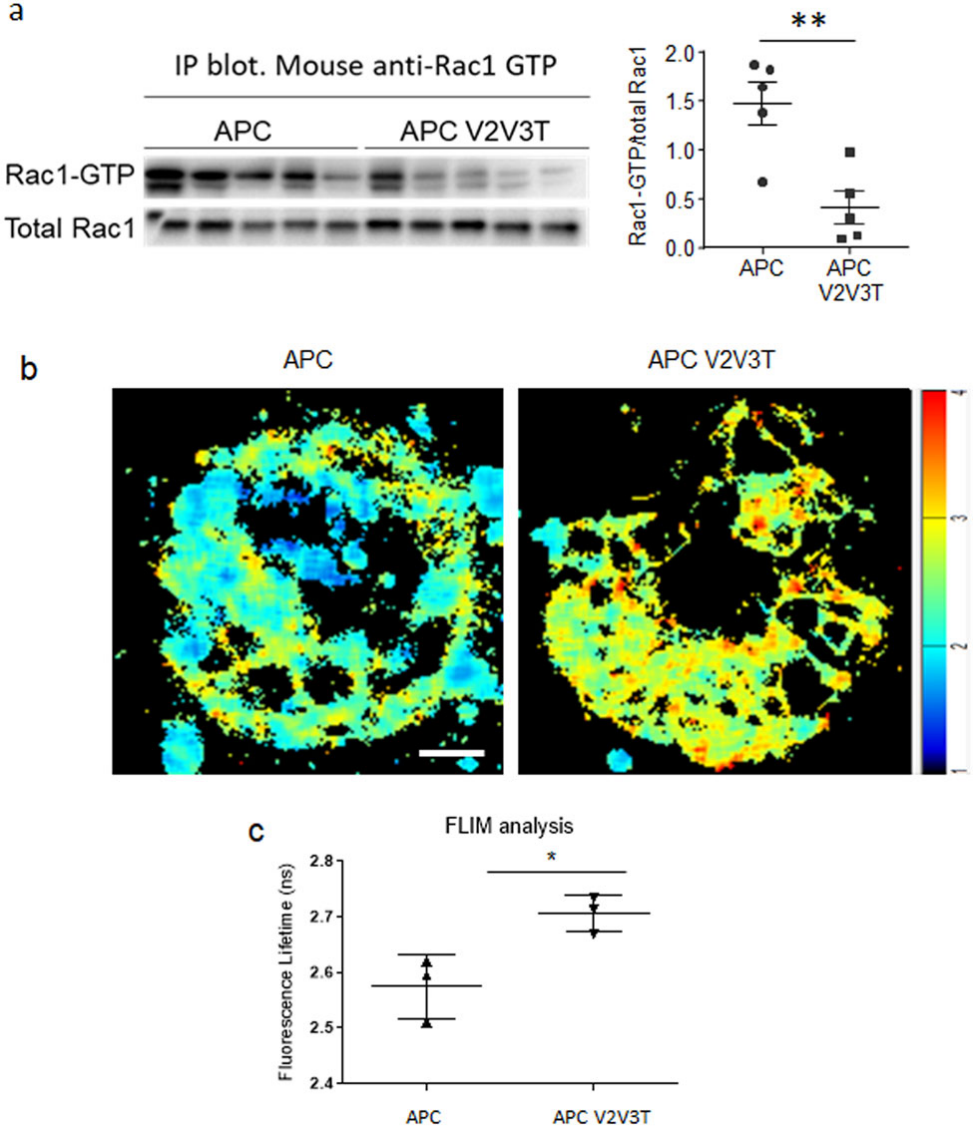
Loss of the triple GEFs results in a downregulation of junctional RAC1 activity. **a** Immunoprecipitation of Active RAC1 from intestinal epithelium, blotted with total RAC1 and the corresponding scoring. *n* = 3 biologically independent samples for *Vil-CreER*^T2^ *Apc*^*fl/fl*^ (APC) and *Vil-CreER*^T2^ *Apc*^*fl/fl*^, *Vav2*^−/−^, *Vav3*^−/−^ *Tiam1*^−/−^ (APC V2V3T). *p = 0.00799 as determined by a one-tailed Mann–Whitney test. Data are presented as mean values ±SD. **b** FLIM-FRET analysis of organoids day 3 post isolation from *Vil-CreER*^*T2*^ *Apc*^*fl/fl*^ (APC) and *Vil-CreER*^*T2*^ *Apc*^*fl/fl*^ *Vav2*^−/−^*, Vav3*^−/−^*, Tiam1*^−/−^ (APC V2V3T) Scale bar represents 20 μm. **c** Quantification of FLIM-FRET analysis at cell–cell contacts. *N* = 3 biologically independent cell lines derived from independent mice for each genotype. *p = 0.0426 as determined a two-tailed unpaired *T*-test. Data are presented as mean values ±SD.

To better understand gross RAC1 activity in APC-deficient intestinal tissue when compared to APC V2V3T intestinal tissues, we performed immunoprecipitation assays with a RAC1-GTP specific antibody. This approach demonstrated that the overall proportion of GTP bound or “active” RAC1 in APC-deficient intestinal tissue is substantially lower in the absence of VAV2, VAV3 and TIAM1 (Fig. 5a). To complement this gross or bulk approach to assessment of RAC1 activity, we took advantage of the previously described genetically engineered mouse expressing a Raichu-RAC1 FRET probe^54,55^. This probe was previously shown to enable the quantification of active RAC1 in live tissues and cells, whilst leaving the cell properties and responses unaffected. Johnsson et al. previously detected Raichu-RAC1 expression in organoid intestinal cultures and showed spatial and temporal regulation of RAC1 activity in these cultures particularly in the stem-cell niche. Given the versatility of this Raichu-RAC1 probe, we crossed the Raichu-*Rac1-*FRET mice to *Vil-CreER*^*T2*^ *Apc*^*fl/fl*^ *Vav2*^−/−^ *Vav3*^−/−^ *Tiam1*^−/−^ mice in order to quantify RAC1 activity caused by APC loss, which is dependent on the triple-GEF expression. In these organoid cultures, we were able to utilise FLIM-FRET imaging to quantify RAC1 activity. This approach indicated a decrease in RAC1 activity in APC V2V3T organoids when compared to APC controls, as demonstrated by an increase in fluorescence lifetime (Fig. 5b, c). We have previously demonstrated that following APC loss in the intestinal epithelium, RAC1 has a role in generation of reactive oxygen species (ROS)^14^. Critically this is a RAC1 specific function, as CDC42 is unable to stimulate ROS generation through NADPH^56^. In the present study, reduced lipid peroxidation indicated by immunohistochemical staining of malondialdehyde (MDA) in the intestinal epithelium of APC V2V3T or APC *Rac1*^*fl/fl*^ when compared to APC controls suggests a suppression of ROS generation in the context of reduced RAC1 activity (Supplementary Fig. 11A, B).

### Oncogenic mutation of KRAS drives resistance to RAC-GEF depletion

**Fig. 6:**
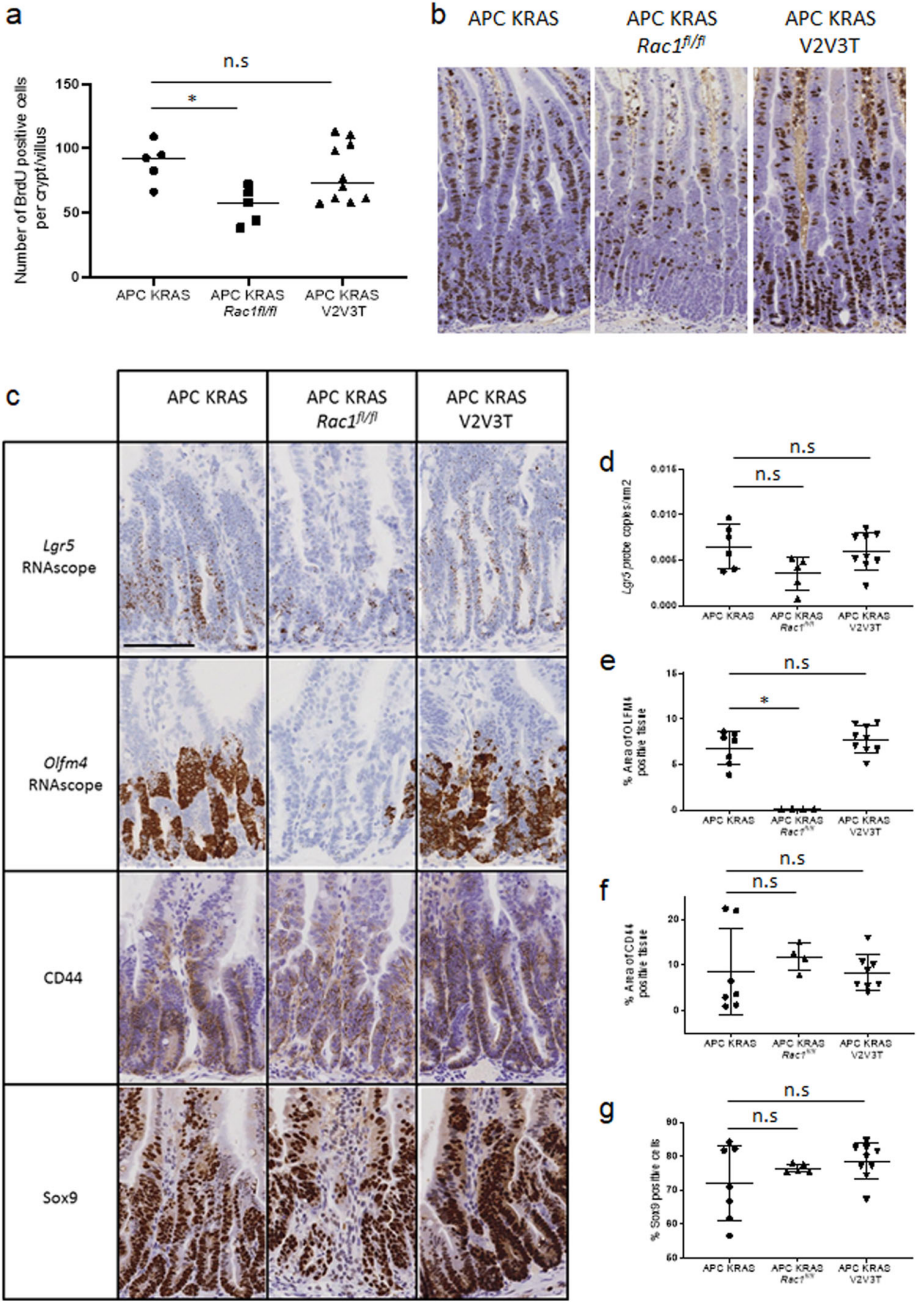
Oncogenic mutation of KRAS drives resistance to RAC-GEF depletion. **a** Quantification of BrdU positive cells. *N* = 5, 5 and 10 biologically independent animals for *Vil-CreER*^T2^ *Apc*^*fl/fl*^ *KRas*^*G12D*^ (APC KRAS), *Vil-CreER*^T2^ *Apc*^*fl/fl*^, *KRas*^*G12D*^*, Rac1*^*fl/fl*^ (APC KRAS *Rac1*^*fl/fl*^) and *Vil-CreER*^T2^ *Apc*^*fl/fl*^, *KRas*^*G12D*^*, Vav2*^−/−^, and *Vav3*^−/−^ *Tiam1*^−/−^ (APC KRAS V2V3T) respectively. APC KRAS vs APC KRAS *Rac1*^*fl/fl*^ *p* = 0.0461 APC KRAS vs APC KRAS V2V3 *p* = 0.9363 as determined by a two-tailed Kruskal–Wallis statistical analysis with Dunn’s mutltiple comparisons test (under the control of *Vil-CreER*^*T2*^). Data are presented as mean values ±SD. **b** Representative images for BrdU incorporation of *Vil-CreER*^T2^ *Apc*^*fl/fl*^ *KRas*^*G12D*^ (APC KRAS), *Vil-CreER*^T2^ *Apc*^*fl/fl*^, *KRas*^*G12D*^*, Rac1*^*fl/fl*^ (APC KRas *Rac1*^*fl/fl*^) and *Vil-CreER*^T2^ *Apc*^*fl/fl*^, *KRas*^*G12D*^*, Vav2*^−/−^ and *Vav3*^−/−^ *Tiam1*^−/−^ (APC KRAS V2V3T). Scale bar represents 100 µm. **c** Representative images of RNAscope for *Lgr5* and *Olfn4* and IHC for CD44 and SOX9 *Vil-CreER*^T2^ *Apc*^*fl/fl*^ *KRas*^*G12D*^ (APC KRAS), *Vil-CreER*^T2^ *Apc*^*fl/fl*^, *KRas*^*G12D*^*, Rac1*^*fl/fl*^ (APC KRAS *Rac1*^*fl/fl*^) and *Vil-CreER*^T2^ *Apc*^*fl/fl*^, *KRas*^*G12D*^*, Vav2*^−/−^ and *Vav3*^−/−^ *Tiam1*^−/−^ (APC KRAS V2V3T). Scale bar represents 100 µm. **d** Quantification of *Lgr5* RNAscope. *Vil-CreER*^T2^ *Apc*^*fl/fl*^ *KRas*^*G12D*^ (APC KRAS vs *Vil-CreER*^T2^ *Apc*^*fl/fl*^, *KRas*^*G12D*^*, Rac1*^*fl/fl*^ (APC KRAS *Rac1*^*fl/fl*^) *p* = 0.1105 APC KRAS vs *Vil-CreER*^T2^ *Apc*^*fl/fl*^, *KRas*^*G12D*^*, Vav2*^−/−^, *Vav3*^−/−^ *Tiam1*^−/−^ (APC KRAS V2V3T). *n* = 6, 5 and 8 biologically independent animals for APC KRAS, APC KRAS *Rac1*^*fl/fl*^, and APC KRAS V2V3T respectively. *p* = >0.999 as determined by a two-tailed Kruskal–Wallis statistical analysis with Dunn’s mutltiple comparisons test (under the control of *Vil-CreER*^*T2*^). Data are presented as mean values ±SD. **e** Quantification of *Olfm4* RNAscope. *Vil-CreER*^T2^ *Apc*^*fl/fl*^ *KRas*^*G12D*^ (APC KRAS) vs *Vil-CreER*^T2^ *Apc*^*fl/fl*^, *KRas*^*G12D*^*, Rac1*^*fl/fl*^ (APC KRAS *Rac1*^*fl/fl*^) *p* = 0.356 APC KRAS vs *Vil-CreER*^T2^ *Apc*^*fl/fl*^, *KRas*^*G12D*^*, Vav2*^−/−^, *Vav3*^−/−^ *Tiam1*^−/−^ (APC KRAS V2V3T). *n* = 6, 4 and 9 biologically independent animals for APC KRAS, APC KRAS *Rac1*^*fl/fl*^ and APC KRAS V2V3T respectively. *p* = 0.9381 as determined by a two-tailed Kruskal–Wallis statistical analysis with Dunn’s mutltiple comparisons test (under the control of *Vil-CreER*^*T2*^). Data are presented as mean values ±SD. **f** Quantification of CD44 IHC. *Vil-CreER*^T2^ *Apc*^*fl/fl*^ *KRas*^*G12D*^ (APC KRAS) vs *Vil-CreER*^T2^ *Apc*^*fl/fl*^, *KRas*^*G12D*^*, Rac1*^*fl/fl*^ (APC KRAS *Rac1*^*fl/fl*^) *p* = 0.2061 APC KRAS vs *Vil-CreER*^T2^ *Apc*^*fl/fl*^, *KRas*^*G12D*^*, Vav2*^−/−^, *Vav3*^−/−^ *Tiam1*^−/−^ (APC KRAS V2V3T). *n* = 7, 4 and 8 biologically independent animals for APC KRAS, APC KRAS *Rac1*^*fl/fl*^ and APC KRAS V2V3T respectively. *p* = >0.999 as determined by a two-tailed Kruskal–Wallis statistical analysis with Dunn’s mutltiple comparisons test (under the control of *Vil-CreER*^*T2*^). Data are presented as mean values ±SD. **g** Quantification of SOX9 IHC. *Vil-CreER*^T2^ *Apc*^*fl/fl*^ *KRas*^*G12D*^ (APC KRAS) vs *Vil-CreER*^T2^ *Apc*^*fl/fl*^, *KRas*^*G12D*^*, Rac1*^*fl/fl*^ (APC KRAS *Rac1*^*fl/fl*^) *p* = >0.999 APC KRAS vs *Vil-CreER*^T2^ *Apc*^*fl/fl*^, *KRas*^*G12D*^*, Vav2*^−/−^, *Vav3*^−/−^ *Tiam1*^−/−^ (APC KRAS V2V3T). *n* = 7, 5 and 9 biologically independent animals for APC KRAS, APC KRAS *Rac1*^*fl/fl*^ and APC KRAS V2V3T respectively *p* = 0.4088 as determined by a two-tailed Kruskal–Wallis statistical analysis with Dunn’s mutltiple comparisons test (under the control of *Vil-CreER*^*T2*^). Data are presented as mean values ±SD.

In this simple model of adenoma formation in the intestinal epithelium, we have observed marked compensation between GEFs at the level of transcription. Critically, both the data described above, and the regulatory complexity generated both transcriptionally and functionally by the expression of 60+ multidomain cellular RAC-GEFs, implies significant plasticity in regulation of GTPase signalling in response to altered cellular or environmental cues. For this reason, we sought to determine whether a further compounding oncogenic KRAS mutation could impact sensitivity to RAC-GEF depletion in vivo. Clinically, KRAS mutant colorectal cancers are refractory to current treatments, and critically, one could envisage that significant alteration to signalling flux through oncogenic pathways controlled by KRAS could have a significant impact upon RAC-GEF and consequently RAC GTPase activity. To investigate this, we intercrossed an oncogenic LSL-*KRas*^G12D^ allele^57^ to the *Vil-Cre*^*ERT2*^ *Apc*^*fl/fl*^ *Rac1*^*fl/fl*^ and *Vil-Cre*^*ERT2*^ *Apc*^*fl/fl*^ *Vav2*^−/−^ *Vav3*^−/−^ *Tiam1*^−/−^ models described both above and previously^14^. This approach allowed us to test the impact of a strong oncogenic drive upon sensitivity to genetic targeting of either *Rac1* alone or a combination of the RAC-GEFs *Vav2*, *Vav3*, *Tiam1*. As previously reported, targeting of oncogenic KRAS to the intestinal epithelium in the context of APC depletion results in exacerbated crypt expansion driven by a significant increase in cellular proliferation^58^. Here, enhanced KRAS driven crypt expansion retained sensitivity to homozygous depletion of RAC1, indicating that in the context of KRAS mutation, RAC1 signalling remains a fundamental determinant of cellular proliferation. Notably, despite the retained sensitivity to RAC1 depletion, the presence of an oncogenic KRAS mutation drove resistance to the combined depletion of VAV2, VAV3 and TIAM1, with no significant reduction in epithelial cell proliferation observed in *Vil-Cre*^*ERT2*^ *Apc*^*fl/fl*^ *Kras*^*G12D/+*^ *Vav2*^−/−^ *Vav3*^−/−^ *Tiam1*^−/−^ when compared to *Vil-Cre*^*ERT2*^ *Apc*^*fl/fl*^ *Kras*^*G12D/+*^ control (Fig. 6a, b). Indeed, in contrast to APC deficiency, depletion of VAV2, VAV3 and TIAM1 had no impact upon intestinal stem marker or WNT target gene expression in KRAS mutant tissue (Fig. 6c–g). Consistent with these observations, RAC1 depletion was sufficient to suppress intestinal stem cell marker expression, suggesting that RAC1 activity may be uncoupled from VAV2, VAV3 and TIAM1 activity in the context of KRAS mutation (Fig. 6c–g). In light of these observations, along with the altered transcriptional regulation of RAC-GEFs driven by APC depletion, and the impact that oncogenic KRAS mutation has upon GTPase signalling at the network level^59^, it is conceivable that KRAS mutation may result in transcriptional control of alternative RAC-GEF molecules. We sought to address this question through comparative transcriptional profiling of RAC-GEF expression in intestinal tissue from *Vil-Cre*^*ERT2*^ *Apc*^*fl/fl*^ and *Vil-Cre*^*ERT2*^ *Apc*^*fl/fl*^ *Kras*^*G12D/+*^ mice. Intriguingly, we found that expression of oncogenic KRAS mutation did not broadly impact the transcription RAC-GEFs (Supplementary Fig. 12). Thus, the lack of phenotypic effect of RAC-GEF deletion following KRAS mutation may be due to a functional rewiring of cellular signalling upstream of RAC1, either through transcriptional adaptation, or via modified signalling flux through individual molecules or networks of RAC-GEFs. While intriguingly, the lack of a broader KRAS driven function in APC-deficient tissue may be to support activity of KRAS dependent signalling processes, a role rendered redundant by oncogenic KRAS mutation. Certainly, these observations suggest a deeper understanding of the impact of KRAS mutation upon RAC1 activity in CRC appears of critical importance.

The observations detailed here serve to underline the importance of RAC1 GTPase signalling in the regulation of growth and survival of transformed cells in vivo. Moreover, the data suggest that the targeting of specific RAC-GEFs should be informed not only by basal expression or transcriptional induction, but also with consideration of basal activity. Whilst we have demonstrated that combined targeting of VAV2, VAV3 and TIAM1 has significant impact upon phenotypes associated with APC deficiency in the context of intestinal disease, we acknowledge that many open questions remain regarding the mechanistic relationship between uncontrolled WNT signalling and activation of these specific molecules. Nonetheless, the data presented go some way to demonstrate that while drugs designed to suppress small GTPase signalling through targeting of specific GEFs may have significant therapeutic impact in early disease, they may ultimately fail in more complex late stage disease, due not only to functional redundancy but also due to inherent plasticity within upstream regulatory networks.

## Materials and methods

### Mouse experiments

All experiments were performed under the UK Home Office Regulations (Owen Sansom PPL 70-8646) in adherence with the ARRIVE guidelines and approved by the University of Glasgow Animal Welfare Ethical Review Board. *Vil-CreER*^T2^ and *Lgr5-EGFP-IRES-creER*^*T2*^ mice of both sexes and of a mixed genetic background were used throughout. Mice were housed in conventional caging with environmental enrichment on a 12-h light–dark cycle with access to food and water ad libitum. Mice were induced between the age of 6 and 12 weeks, once they had reached a minimum weight of 20 g and were assessed for symptoms of ill health at least 3 times per week. The alleles used for this study were as follows; *Vil-CreER*^T2^^60^, *Lgr5-EGFP-IRES-creER*^*T2*^^61^, *Apc*^*580S*^^62^, *Kras*^*LSL-G12D*^^57^, *Rac1*^*fl*^^63^, *Vav2*^−/−^^64^, *Vav3*^−/−^^65^, *Tiam1*^−/−^^34^, *LifeAct*-GFP^42^ and *Raichu-Rac1*^54^. Recombination by *Vil-CreER*^T2^ was induced by a single intraperitoneal (IP) injection of tamoxifen (80 mgkg^−1^) per day for 2 days in the context of *Apc*^*fl/fl*^ animals, and 1 day in the case of *Apc*^*fl/fl*^; *Kras*^*LSL-G12D/+*^ animals. Tumourigenicity studies were carried out using *Lgr5-EGFP-IRES-creER*^*T2*^, and mice were induced by one IP injection of 120 mg/kg tamoxifen followed by 3 daily doses of 80 mg/kg. Mice were aged until they reached endpoint (pale feet and weight loss). AOM-DSS colitis-associated CRC was generated through one IP injection of 12 mg/kg AOM (Sigma Aldrich), 5 days after which 2.5% DSS (MP Biomedicals, MQ 36-50kDA) was given orally in the drinking water for 5 days, followed by 16 days of normal water. This DSS cycle was carried out a total of three times after which the mice were killed and samples taken for histological analysis. The liver work was carried out following Cre-mediated recombination using AAV8.TBG.Pi.Cre.rBG^53^. Viral induction was administered through tail vain induction at a concentration of 2.5 × 10^11^ copies/ml. Mice were killed 8 days post induction and livers and serum were taken for analysis.

### Immunohistochemistry

All immunohistochemistry was carried out using samples fixed in 10% neutral-buffered formalin for 12–18 h at 4 °C prior to paraffin embedding. Antigen retrieval was performed using citrate pH 6 heat mediated antigen retrieval. Primary antibodies and concentrations were used as follows; VAV1 (1:50; Cell Signalling #2502), RAC1 (phospho S71) (1:500; Abcam Ab203884); CDC42 (1:200; Abcam Ab155940);^66^ Active RAC1 (1:500, NewEast Biosciences; cat# 26903)^67^, Cleaved caspase-3 (1:500; Cell Signalling; cat# 9661)^68^, E-Cadherin (clone 24E10, 1:200; Cell Signalling cat#3195)^69^, SOX9 (1:500; Millipore; cat# AB5535), β-catenin (1:50; BD Biosciences; cat#610154)^70^, CD44 (1:100; BD Biosciences; cat# 550538)^71^, MDA (1:100; Abcam Ab6463);^13^ VAV2 (1:200; Abcam Ab79182); VAV3 (1:200; Abcam; Ab203315); CTGF (1:200; Abcam; Ab6992)^48^. Secondary HRP-tagged secondaries were used as follows; Dako Envision+ system HRP goat anti-mouse (neat; Dako K4001), Dako Envision+ system HRP goat anti-rabbit (neat; Dako K4003). Secondary fluorescent antibodies were used as follows; Alexafluor-488 (1:200; ThermoFisher; cat # A11034) and AlexaFluor 594 (1:200; ThermoFisher; cat #A11032). Proliferation was determined by quantifying BrdU incorporation (1:500; BD Biosciences Cat # 347580).

RNAscope probes; *Vav2* (ACD 437428), *Lgr5* (ACD 312178), *Olfm4* (ACD 311838), *c-Myc* (ACD 413458) and *Axin2* (ACD 400338) were 2.5 LS probes obtained from ACD, and were stained using the 2.5LS reagent kit-brown (ACD 322100) on the Leica Bond Rx autostainer. BaseScope Probe; *Rac1* (ACD 710331) was stained using the BaseScope detection reagents red kit (322910) following the manufacturers’ instructions.

### Tumour burden analysis

Using *Lgr5-EGFP-IRES-creER*^*T2*^ to drive APC loss results in a lawn of microscopic tumours as opposed to macroscopic tumours. As such, we used Halo v 3.1.1076.363 (Indica labs) to microscopically determine the total area of tissue and the percentage of this that is composed of lesions and tumours.

### Consensus molecular sequencing analysis

CRC patient expression data (Illumina HiSeq, *N* = 326) was obtained from the TCGA project using the FIREHOSE repository (https://gdac.broadinstitute.org/). CMS labels for the patients were obtained from Guinney et al.^47^ *P* values were calculated using the limma R package^72^ and Benjamini–Hochberg multiple testing correction was applied^72,73^.

### Assessment of RAC1 activity through immunoprecipitation of RAC1-GTP

RAC1 activity was determined on snap frozen small intestinal tissue and performed using the Cytoskeleton Rac1-activation assay kit (Cytoskeleton; #BK035) according to the manufacturer’s instructions. Briefly, tissues were lysed with lysis buffer supplemented with protease inhibitors. Around 800 μg of lysates was precleared and PAK-PBD beads were added to pull-down active Rac1. Positive (GTPγS) and negative (GDP) bound control samples were prepared according to standard protocol (Cytoskeleton). Following immunoprecipitation at 4 °C for 1 h, beads were washed 3x in washing buffer, and precipitated proteins detected by polyacrylamide gel electrophoresis and subsequent western blotting. Protein bound membranes were incubated overnight at 4 °C together with primary anti-Rac1 monoclonal antibody (Cytoskeletion; #ARC03; 1:500) in block solution (TBS containing 5% BSA, and 0.02% Triton X-100). After washing the membranes thoroughly in TBS-T, they were incubated 1 h at room temperature with secondary goat mouse antibody (Agilent #P044701-2, 1:2000) in blocking solution. For assessment of total RAC1 in whole cell lysates (20 μg protein/sample), mouse anti-RAC1 antibody (Cytoskeletion; #ARC03; 1:500) was used.

### RNA isolation

RNA was isolated from mouse intestinal tissue collected 5 cm distal from the stomach. RNA isolation from whole intestinal tissue required dissociation using a Precellys 24 homogeniser (Bertin), and RNA was isolated using a Qiagen RNeasy Mini Kit (Qiagen, Crawley, West Sussex, UK), according to the manufacturer’s instructions. RNA isolation from organoids required isolation of organoids (detailed below) from intestinal tissue. Cells were cultured for 3 days and then snap frozen. RNA was then extracted using a Qiagen RNeasy Mini Kit. In both cases, DNAse treatment was performed using Qiagen DNAse (cat #72954).

### RNA-sequencing library generation and sequencing

The quality and quantity of purified RNA was determined using an Agilent 2200 Tapestation with RNA screentape (Agilent, ThermoFisher Scientific) (RIN of at least 8 was required). RNA-seq libraries were generated as described in TruSeq RNA Sample Preparation Guide (illumina, part no. RS-122-2001) using Illumina TruSeq RNA LT sample preparation kit. PolyA selection step was performed on 1 µg of total RNA; followed by an 8 min heat fragmentation step aimed at producing libraries with an insert size between 120 bp and 200 bp. First strand cDNA was synthesised from the enriched and fragmented RNA using SuperScript III Reverse Transcriptase (Invitrogen, 18080-044) and random primers. Second strand synthesis was performed to produce ds cDNA. Following end repair; 3′ adenylation and ligation of adaptors to the dsDNA was performed; libraries were subjected to 13 cycles of PCR. RNA-seq libraries were quantified using Qubit v2.0 HS DNA assay (Invitrogen, Q32854) and sized and qualified using an Agilent 2200 TapeStation with Agilent D1000 ScreenTape (Agilent, 5067-5582). Libraries were normalised to 4 nM and pooled before clustering and sequencing (36 bp paired-end) on a NextSeq500 sequencer (illumina)^74^.

The libraries were run on an Illumina Next Seq instrument using the High Output 75 cycles kit (2 × 36 cycles, paired end reads, single index). Fastq files are deposited at Gene Expression Omnibus (NCBI), study accession numbers; GSE112226 and GSE152388.

### RNA-sequencing analysis

Raw sequence quality was assessed using the FastQC algorithm version 0.11.8, then sequences were trimmed to remove adaptor sequences and low-quality base calls, defined as those with a Phred score of <20, using the Trim Galore tool version 0.6.4. Trimmed sequences were aligned to mouse genome build GRCm38.98 using HISAT2 version 2.1.0, then raw counts per gene were determined using FeatureCounts version 1.6.4. Differential expression analysis was performed using the R package DESeq2 version 1.22.2, and principal component analysis was performed using R base functions.

### Biochemical analysis of Serum

Blood was obtained post-mortem by cardiac puncture at 8 days post induction with AAV8.TBG.Pi.Cre.rBG viral injection. Blood was collected into a microcentrifuge tube and allowed to clot at room temperature. The samples were then centrifuged at 10,000 × *g* for 10 min at 4 °C and the serum collected. This serum was analysed on the Siemens Dimension Expand clinical chemistry system using the IFCC parameters.

### Crypt isolation

Organoids were isolated from murine intestines 4 days post induction. The proximal 10 cm of the small intestine was opened longitudinally and washed with PBS. This proximal intestine was scraped using a coverslip to remove and discard the villi and subsequently cut into small (5 mm) pieces. These pieces were washed with ice-cold PBS and subsequently incubated in 2 mM EDTA in PBS and incubated at 4 °C for 30 min. The tissue was washed with ice-cold PBS with washes 2–4 being collected (crypt enriched fractions). These fractions were diluted with Advanced DMEM/F12 and centrifuged (1200 rpm for 5 min). The pellets were resuspended in Advanced DMEM/F12 and passed through a 70-µm cell strainer^75^. Roughly 100 crypts were plated in 30 μl Matrigel (BD Bioscience) in a 24-well plate with Advanced DMEM/F12 supplemented with 50 ng/µl EGF (Peprotech) and 100 ng/µl Noggin (Peprotech). For clonogenicity assays, established cultures were dissociated to single cells by resuspending a cell pellet in 1 ml of TripLE (Gibco by LifeTechnologies), 100 µl DNase buffer and 10 µl DNase enzyme (Qiagen), incubated for 1 h at 37 °C (or until a single-cell suspension was achieved). The cells were passed through a 40 µl cell strainer and counted. Cells were plated at 10,000 cells/well in a 24-well plate and grown for 5 days. After 5 days, established organoids were counted and numbers normalised to *Vil-CreER*^T2^ *Apc*^*fl/fl*^ cultures, where three biological replicates and at least three technical replicates were used per genotype.

### Scanning electron microscopy

Tissue samples were fixed in Karnovskys fixative for 24 h at 4 °C then rinsed and stored in 0.1 M cacodylate buffer. The specimens were then dehydrated by immersion in a graded series of acetones (30%, 50%, 70%, 80%, 90%, 95% and 100%) each for 5 min. Critical point drying was carried out using an EMS 850 Critical Point Dryer (EMS, Hatfield, UK). Once dry, the tissues were mounted on aluminium stubs using quick drying silver conductive adhesive paint then coated with gold/palladium using an Emscope SC500A sputter coater. The specimens were viewed using a Hitachi S-570 Scanning electron microscope at an operating voltage of 15 kV and working distance of 12. Images were captured using a high resolution image capture system linked to the SEM (Raith GmbH, Germany).

### In-vivo imaging

Intestinal tissue sections were taken from the *Vil-CreER*^T2^*Apc*^*fl/fl*^, *LifeAct*-GFP mice lacking VAV2, VAV3 and TIAM1 or appropriate controls. Tissue was placed either crypt up or villus up on a 20-mm glass-bottom microwell dish (MatTek) with a drop of PBS on top to prevent the tissue from drying out. Imaging was carried out on LaVision Biotech TRIMscope multiphoton microscope, obtaining Z-stacks of 400 μm at 0.4 μm intervals. A Coherent Chameleon Ultra II Ti:Sapphire laser was used at 890 nm to generate fluorescence and second harmonic signal simultaneously. A sequence of dichroic and band-pass filters were employed to spectrally separate the emission. A 500 LP dichroic (Chroma 500dclp) reflects the SHG signal through a Semrock 435/40 nm band-pass filter and transmits through to a Semrock 525/50 nm band-pass filter for the GFP emission. An exponential power increase was applied automatically with increasing focusing depth to counteract the tissue induced scattering of the light. At least three mice were imaged for each genotype.

### FLIM-FRET

Organoids derived from the intestinal epithelium of *Vil-CreER*^T2^ *Apc*^*fl/fl*^ and *Vil-CreER*^*T2*^ *Apc*^*fl/fl*^ *Vav2*^−/−^ *Vav3*^−/−^ *Tiam1*^−/−^ mice were isolated as described above^75^. Three days post isolation, the organoids were imaged on a LaVision Biotec TRIMscope multiphoton microscope using the same setup as used for *LifeAct*-GFP imaging, however, the PMT used to image the GFP emission was replaced with a Hamamatsu hybrid-PMT operating in a photon counting mode to act as TCSPC FLIM detector. Care was taken to use laser power low enough as to not cause photon pile-up, but sufficient to generate signal for accurate curve fitting. Analysis was performed using the built-in non-linear curve fitting routine in the Imspector package from LaVision Biotec (version 7_0_153), and briefly, a tail fitting method was used to exclude the excitation peak and fit a single exponential decay to the fluorescence decay stored at each pixel separately. The decay lifetime was then recorded on a cell by cell basis. At least five cell contacts per organoid were analysed, with at least four organoids per cell line imaged, and separate cell lines generated from three mice of each genotype.

### Statistics and reproducibility

All statistical analyses were carried out using GraphPad Prism V7.02. The Statistical tests used are indicated where appropriate in figure legends. Power analyses were calculated using G* power v3.1.9.4. For *p* values where >0.999 is stated, this is considered as an exact value by Prism. All statistical tests have been mentioned in their respective legends, where they can be sided this has been stated.

For all in vivo analysis each data point represents a biologically unique animal. The clonogenecity and FLIM-FRET experiments were comprised of three biologically distinct replicates and at least three technical replicates.

### Reporting summary

Further information on research design is available in the Nature Research Reporting Summary linked to this article.

## Supplementary information

Supplementary Information

Reporting Summary

## Data Availability

RNAseq data that support the findings in this manuscript have been deposited at Gene Expression Omnibus (NCMI) with the study accession codes; GSE112226 and GSE152388. Data for the consensus molecular sequencing analysis, CRC patient expression data was obtained from the TCGA project using the FIREHOSE repository (https://gdac.broadinstitute.org/). Source data are provided with this paper.

## Source data

Source data
