# Supplementary material for: A RAC-GEF network critical for early intestinal tumourigenesis
Source: Nat Commun. 2021 Jan 4;12:56. doi: 10.1038/s41467-020-20255-4 (PMC7782582; doi:10.1038/s41467-020-20255-4)
Supplement: Supplementary file 1 — Supplementary Information [file 41467_2020_20255_MOESM1_ESM.pdf]

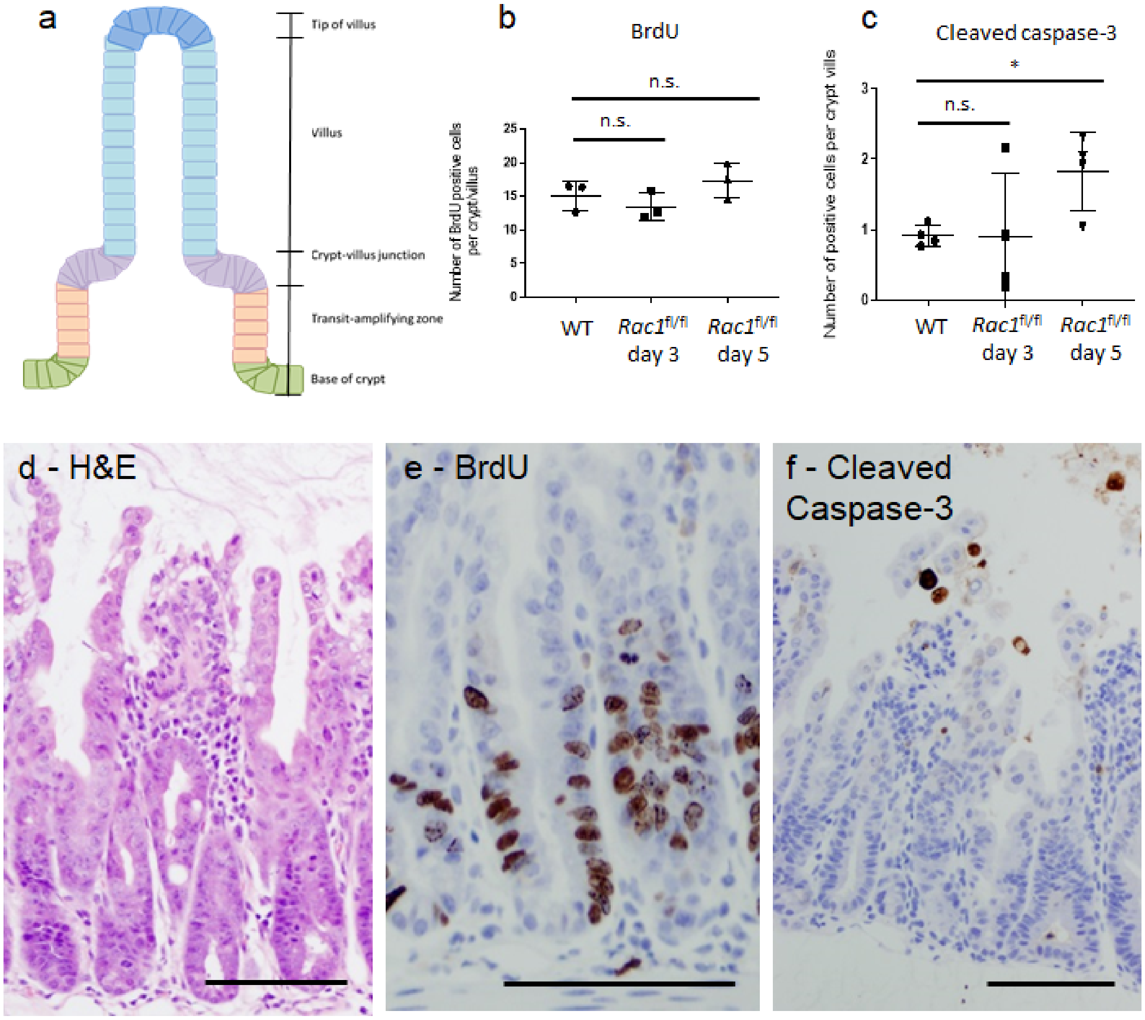

Supplementary Figure 1. Loss of RAC1 perturbs homeostasis of the intestinal epithelium

A) Schematic drawing of the intestinal crypt-villus structure.

B) Analysis of BrdU incorporation in wild-type mice (n = 3 biologically independent animals), or *Vil-CreER*^{T2}, *Rac1*$^{fl/fl}$ (*Rac1*$^{fl/fl}$) either 3 (n = 3 biologically independent animals, p=0.100 as determined by a one-tailed Mann-Whitney) or 5 (n = 4 biologically independent animals, p=0.2 as determined by a one-tailed Mann-Whitney) days post induction. Data are presented as mean values +/- SD.

C) Immunohistochemical analysis of Caspase-3 in wild-type (n = 4 biologically independent animals), or *Vil-CreER*^{T2}, *Rac1*$^{fl/fl}$ (*Rac1*$^{fl/fl}$) mice either 3 (n = 4 biologically independent animals, p=0.3174 as determined by a one-tailed Mann-Whitney) or 5 (n = 5 biologically independent animals, p=0.0286 as determined by a one-tailed Mann-Whitney) days post induction. Data are presented as mean values +/- SD.

D, E, F) Expanded images from Figure 1A of D) H&E, E) BrdU incorporation and F) cleaved-caspase 3 in *Vil-CreER*^{T2}, *Rac1*$^{fl/fl}$ (*Rac1*$^{fl/fl}$) mice, 5 days following induction. Scale bar represents 100 µm in each case.

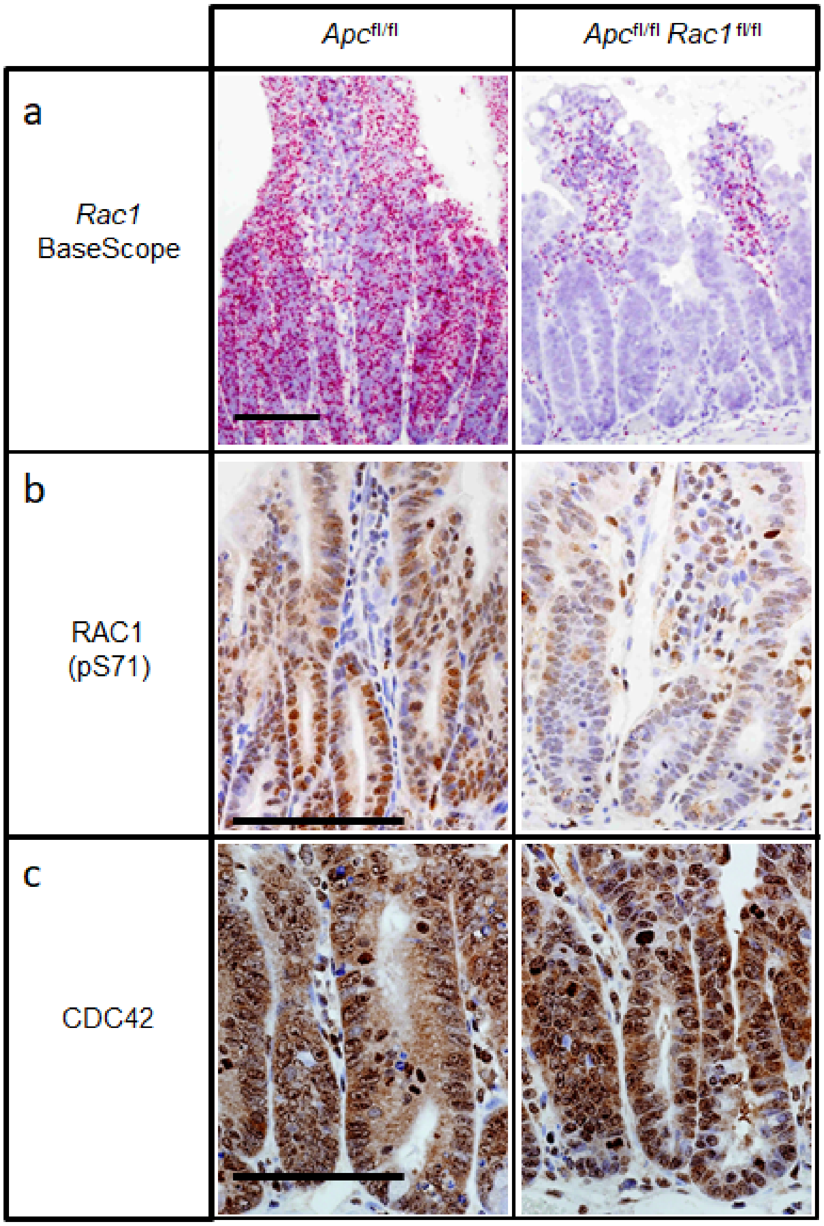

Supplementary Figure 2. Gene and protein analysis of the GTPases RAC1 and CDC42 in the intestinal epithelium

A) BaseScope showing *Rac1* expression in *Vil-CreER*^{T2} *Apc*$^{fl/fl}$ vs *Vil-CreER*^{T2} *Apc*$^{fl/fl}$ *Rac1*$^{fl/fl}$ intestines. Scale bar represents 100 µm.
B) Immunohistochemical analysis of RAC1 (pS71) in *Vil-CreER*^{T2} *Apc*$^{fl/fl}$ vs *Vil-CreER*^{T2} *Apc*$^{fl/fl}$ *Rac1*$^{fl/fl}$ intestines. Scale bar represents 100 µm.
C) Immunohistochemical analysis of CDC42 in *Vil-CreER*^{T2} *Apc*$^{fl/fl}$ vs *Vil-CreER*^{T2} *Apc*$^{fl/fl}$ *Rac1*$^{fl/fl}$ intestines. Scale bar represents 100 µm.

a b

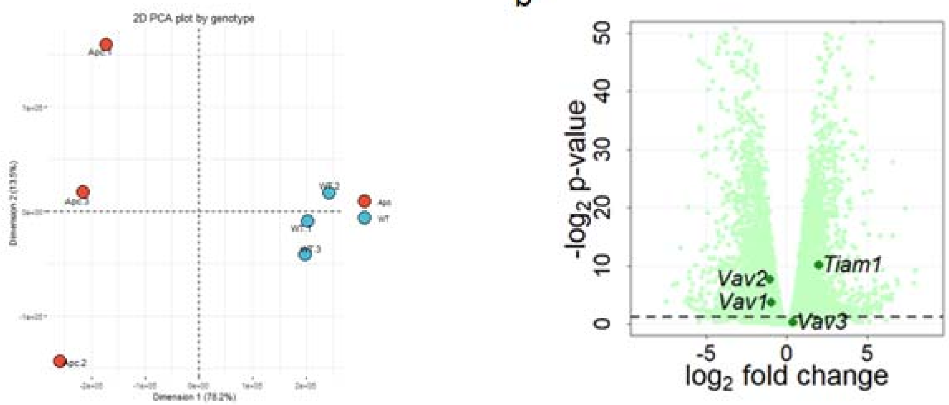

c

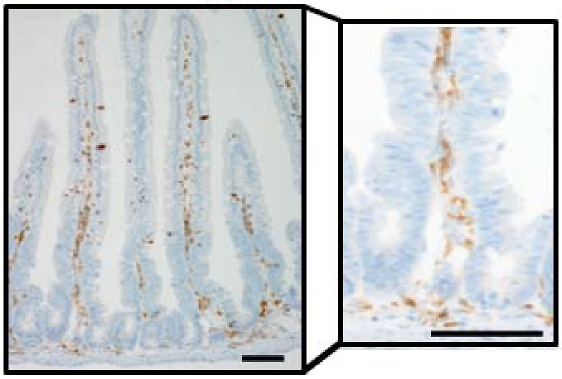

d

Clustal analysis based on amino acid sequence

	VAV2	VAV1	VAV3
VAV2	100.00	53.40	56.42
VAV1	53.40	100.00	58.71
VAV3	56.42	58.71	100.00

e

Clustal analysis based on gene coding sequence

	VAV2	VAV1	VAV3
VAV2	100.00	58.64	58.93
VAV1	58.64	100.00	60.99
VAV3	58.93	60.99	100.00

Supplementary Figure 3. GEF expression from RNA-seq analysis

A) PCA plot of the Wild-type (*Vil-CreER*^{T2}) vs *Vil-CreER*^{T2} *Apc*$^{fl/fl}$ intestine RNAseq analysis.

B) Volcano plot of the RNAseq analysis showing the placement of GEFs of interest within the dataset.

C) Immunohistochemical analysis of VAV1 in intestine from a wild type mouse. Scale bars represent 100 µm in each panel.

D) Clustal analysis of amino acid sequence (Accession numbers; VAV1 NP_0.35821, VAV2 NP_0.33526, VAV3 NP_0.65251)

E) Clustal analysis of gene coding sequence (Accession numbers; VAV1 CT010321, VAV2 U37017)

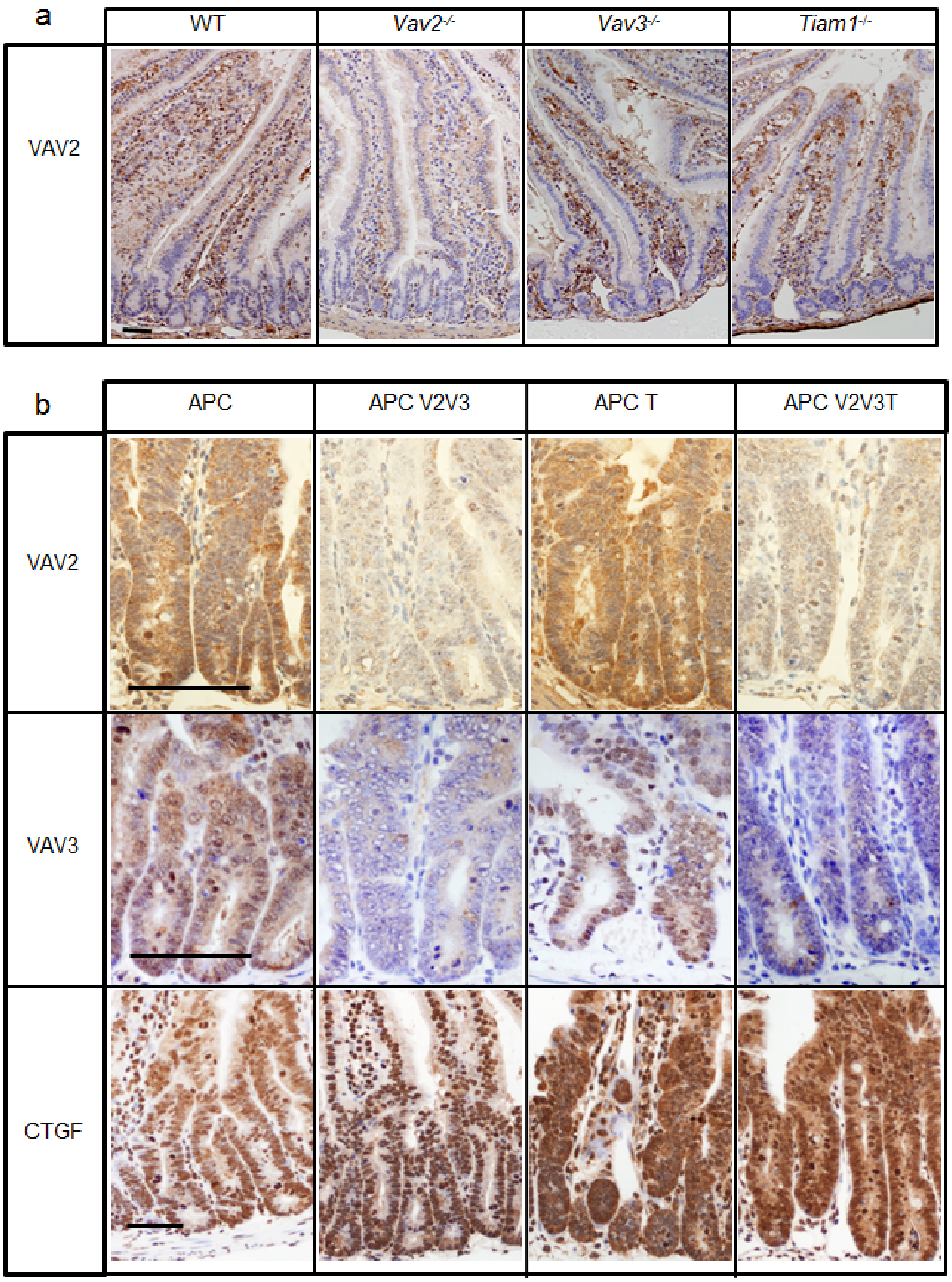

Supplementary Figure 4. Immunohistochemical analysis of VAV2, VAV3 and TIAM1 in the intestinal epithelium.

A) Immunohistochemical analysis of VAV2 in intestines from wild-type (WT), *Vav2*$^{-/-}$, *Vav3*$^{-/-}$ and *Tiam1*$^{-/-}$ mice. Scale bar represents 100 µm

B) Immunohistochemical analysis of VAV2, VAV3 and CTGF (as a marker of TIAM1 activity) in intestines from *Vil-CreER*^{T2} *Apc*$^{fl/fl}$ (APC), *Vil-CreER*^{T2} *Apc*$^{fl/fl}$ *Vav2*$^{-/-}$ *Vav3*$^{-/-}$ (APC V2V3), *Vil-CreER*^{T2} *Apc*$^{fl/fl}$ *Tiam1*$^{-/-}$ (APC T) and *Vil-CreER*^{T2} *Apc*$^{fl/fl}$ *Vav2*$^{-/-}$, *Vav3*$^{-/-}$, *Tiam1*$^{-/-}$ (APCV2V3T) mice. Scale bar represents 100 µm.

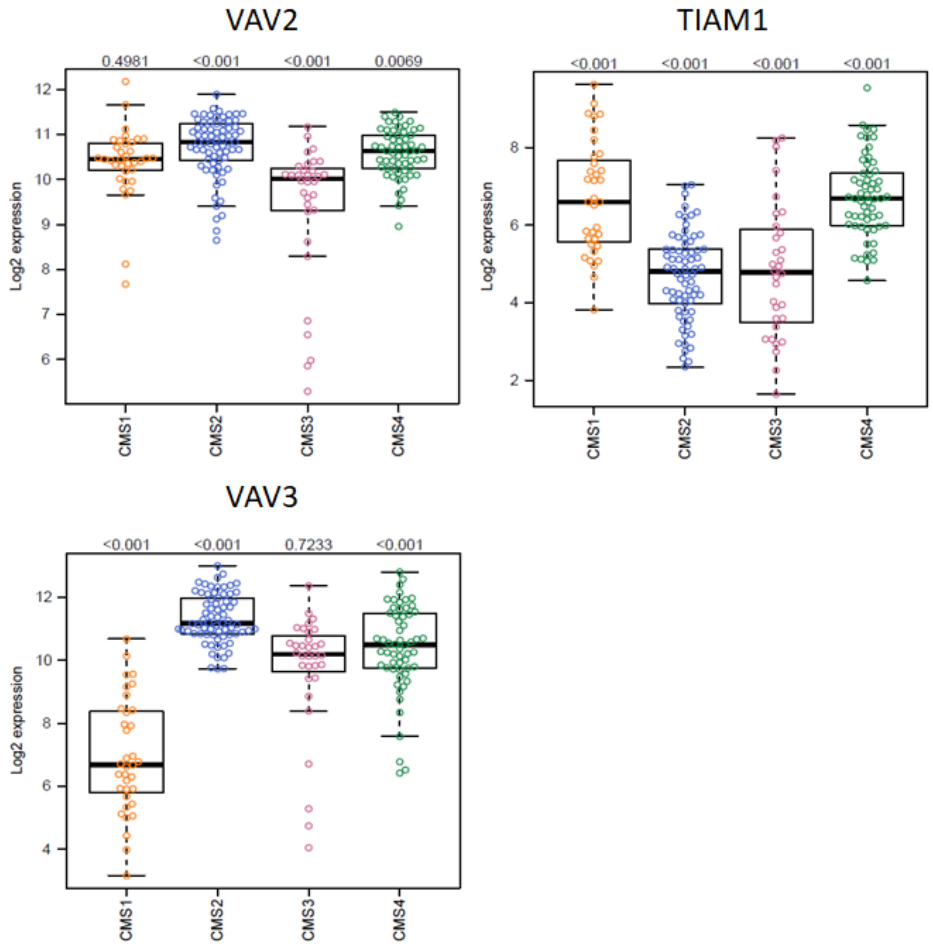

Supplementary Figure 5. Consensus Molecular Subtypes

Relative enrichment of *Vav2*, *Vav3* and *Tiam1* in consensus molecular subtypes of human colorectal cancer generated from TCGA dataset (Illumina HiSeq n=326 biologically independent patient samples). Statistical analysis was performed using limma R package and Benjamini-Hochberg multiple testing correction applied. The boxes indicate inter-quartile range (IQR), with the horizontal black lines representing the median. The whiskers range to a maximum of 1.5 times IQR. Data points outside 1.5IQR are represented by individual dots.

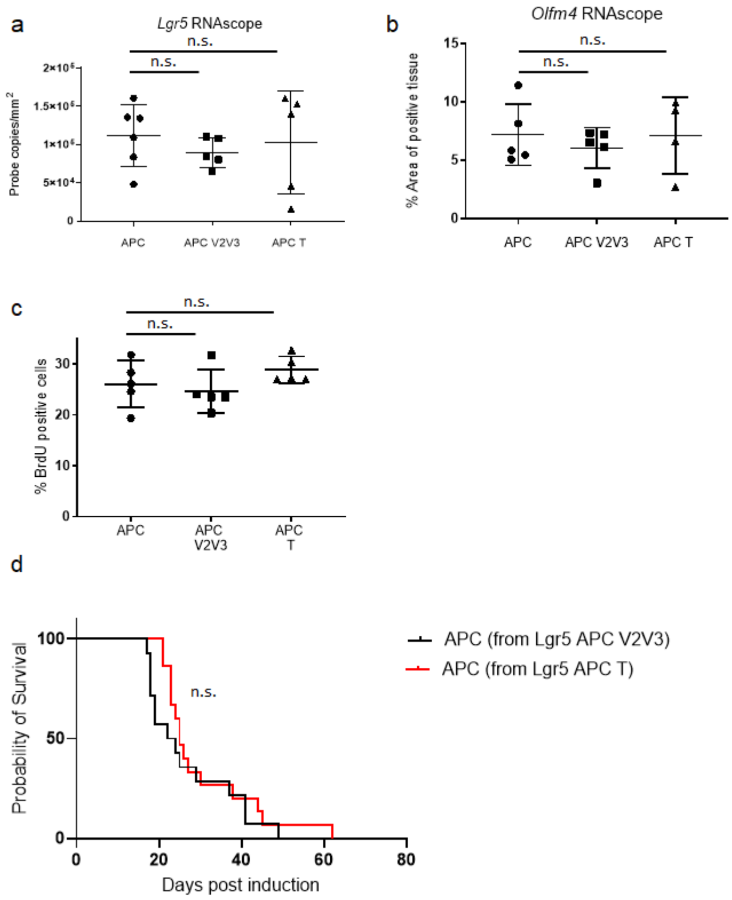

Supplementary Figure 6. Stem cells and proliferation are unchanged by loss of VAV2 and VAV3, or loss of TIAM1.

A) Quantification of *Lgr5* RNAscope.. *Vil-CreER*^{T2} *Apc*$^{fl/fl}$ (APC) (n=6 biologically independent animals) vs *Vil-CreER*^{T2} *Apc*$^{fl/fl}$ *Vav2*$^{-/-}$ *Vav3*$^{-/-}$ (APC V2V3) (n=5 biologically independent animals) p = 0.4786. APC vs *Vil-CreER*^{T2} *Apc*$^{fl/fl}$ *Tiam1*$^{-/-}$ (APC T) (n=5 biologically independent animals) p=0.3008 as determined by a two-tailed Kruskal-Wallis statistical analysis with Dunn's multiple comparisons test (under the control of *Vil-CreER*^{T2}). N indicates biologically independent animals. Data are presented as mean +/- SD.

B) Quantification of *Olfm4* RNAscope. *Vil-CreER*^{T2} *Apc*$^{fl/fl}$ (APC) (n=5 biologically independent animals) vs *Vil-CreER*^{T2} *Apc*$^{fl/fl}$ *Vav2*$^{-/-}$ *Vav3*$^{-/-}$ (APC V2V3) (n=5 biologically independent animals) and APC vs *Vil-CreER*^{T2} *Apc*$^{fl/fl}$ *Tiam1*$^{-/-}$ (APC T) (n=4 biologically independent animals) >0.9999 as determined by a two-tailed Kruskal-Wallis statistical analysis with Dunn's multiple comparisons test (under the control of *Vil-CreER*^{T2}). Data are presented as mean +/- SD.

C) Quantification of BrdU incorporation in end-point tumours. N = 5 biologically independent animals. *Lgr5-EGFP-IRES-creER*^{T2} *Apc*$^{fl/fl}$ (APC) vs *Lgr5-EGFP-IRES-creER*^{T2} *Apc*$^{fl/fl}$ *Vav2*$^{-/-}$, *Vav3*$^{-/-}$ (APC V2V3) p=0.7923. APC vs *Lgr5-EGFP-IRES-creER*^{T2} *Apc*$^{fl/fl}$, *Tiam1*$^{-/-}$ (APC T) p=0.5777 as determined by a two-tailed Kruskal-Wallis statistical analysis with Dunn's multiple comparisons test (under the control of *Vil-CreER*^{T2}*)*. Data are presented as mean +/- SD.

D) Lgr5 APC controls of two ageing cohorts, showing no discernible difference. N= 19 biologically independent animals (Lgr5APC from *Lgr5-EGFP-IRES-creER*^{T2} *Apc*$^{fl/fl}$ V2V3 cohort), n=15 biologically independent animals (Lgr5 APC from *Lgr5-EGFP-IRES-creER*^{T2} *Apc*$^{fl/f}$ T cohort). P= 0.5221 as determined by a two-tailed Log-rank (Mantel-Cox) test.

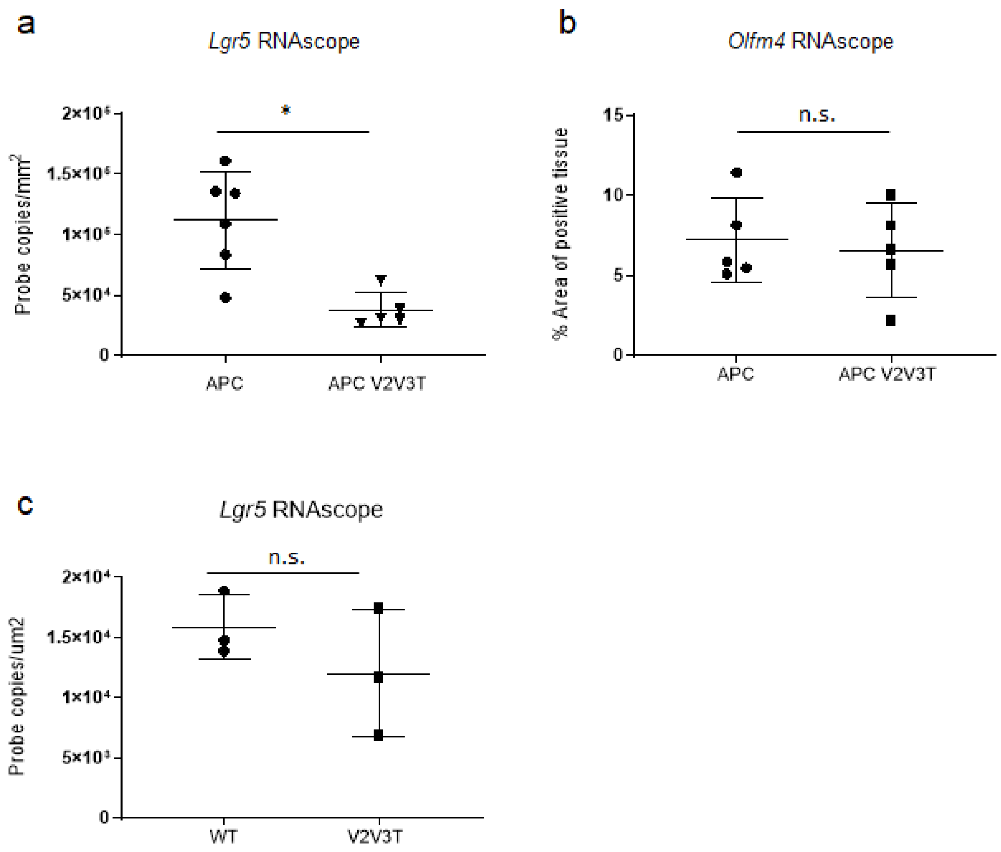

Supplementary Figure 7. Stem cell expression is supressed following deletion of VAV2, VAV3 and TIAM1.

A) Quantification of *Lgr5* RNAscope expression in *Vil-CreER*^{T2} *Apc*$^{fl/fl}$ (APC) vs *Vil-CreER*^{T2} *Apc*$^{fl/fl}$, *Vav2*$^{-/-}$, *Vav3*$^{-/-}$ *Tiam1*$^{-/-}$ (APC V2V3T). APC (n=6 biologically independent animals) vs APC V2V3T (n=5 biologically independent animals) *p = 0.0087 as determined by a two-tailed Mann-Whitney statistical analysis test (under the control of *Vil-CreER*^{T2}*)*. Data are presented as mean +/- SD.

B) Quantification of *Olfm4* RNAscope in *Vil-CreER*^{T2} *Apc*$^{fl/fl}$ (APC) (n=5 biologically independent animals) vs *Vil-CreER*^{T2} *Apc*$^{fl/fl}$, *Vav2*$^{-/-}$, *Vav3*$^{-/-}$ *Tiam1*$^{-/-}$ (APC V2V3T) (n = 5 biologically independent animals), >0.9999 as determined by a two-tailed Mann-Whitney statistical analysis test (under the control of *Vil-CreER*^{T2}*)*. Data are presented as mean +/- SD.

C) Quantification of *Lgr5* RNAscope expression in intestines from wild-type (WT) or *Vav2*$^{-/-}$, *Vav3*$^{-/-}$, *Tiam1*$^{-/-}$ (V2V3T) animals. n=3 biologically independent animals, p=0.400 as determined by a two-tailed Mann-Whitney statistical Analysis test. Data are presented as mean +/- SD.

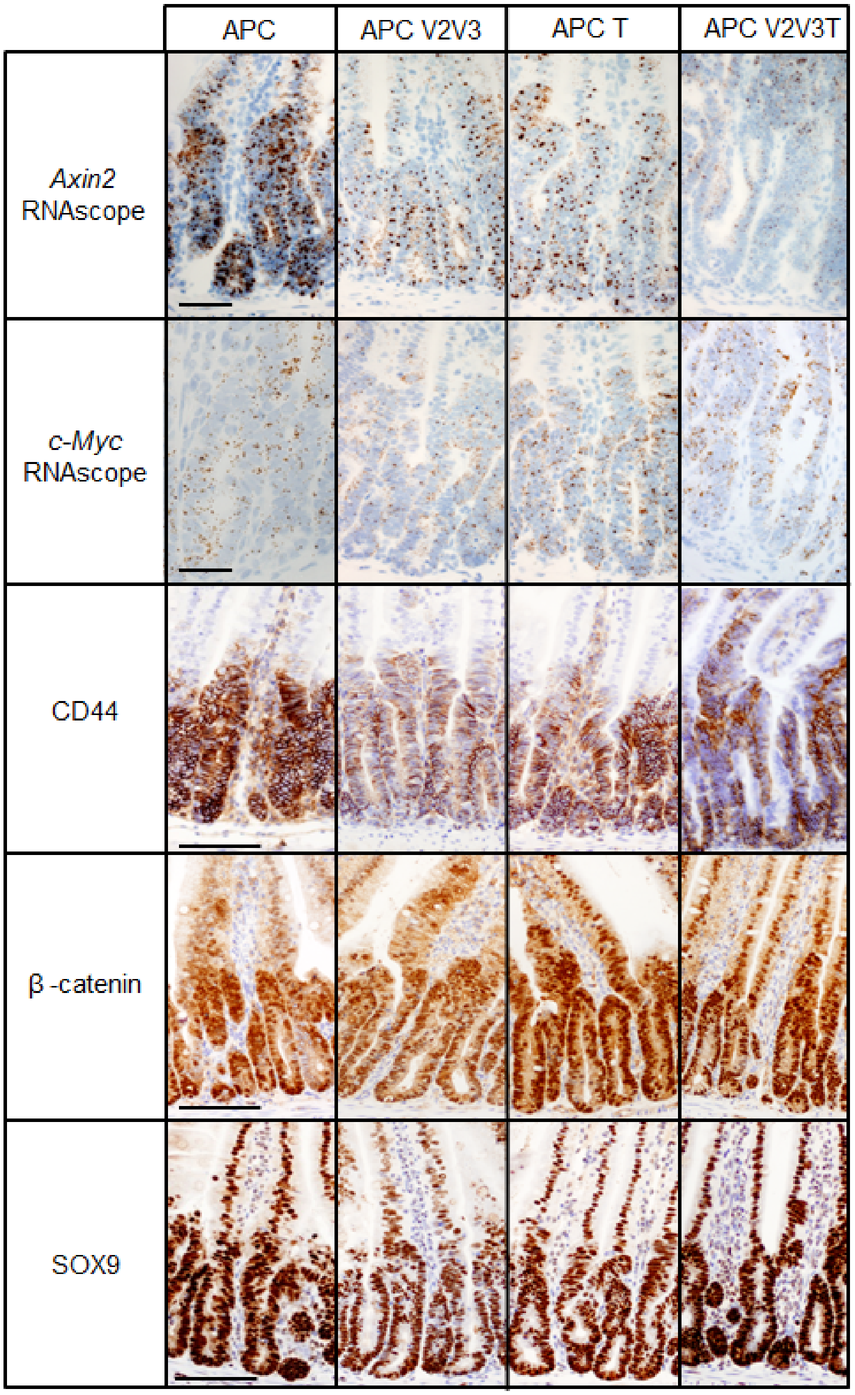

Supplementary Figure 8. WNT target genes are affected following loss of VAV2, VAV3 and TIAM1.

Images of RNAscope of *Axin2* and *c-Myc* and immunohistochemical analysis for CD44, β-catenin and Sox9 on *Vil-CreER*^{T2} *Apc*$^{fl/fl}$ (APC), vs *Vil-CreER*^{T2} *Apc*$^{fl/fl}$, *Vav2*$^{-/-}$, *Vav3*$^{-/-}$ (APC V2V3), vs *Vil-CreER*^{T2} *Apc*$^{fl/fl}$, *Tiam1*$^{-/-}$ (APC T) and vs *Vil-CreER*^{T2} *Apc*$^{fl/fl}$, *Vav2*$^{-/-}$, *Vav3*$^{-/-}$ *Tiam1*$^{-/-}$ (APC V2V3T) (under the control of *Vil-CreER*^{T2}). Scale bar represents 50 µm for RNAscope images and 100 µm for β –catenin, CD44 and Sox9.

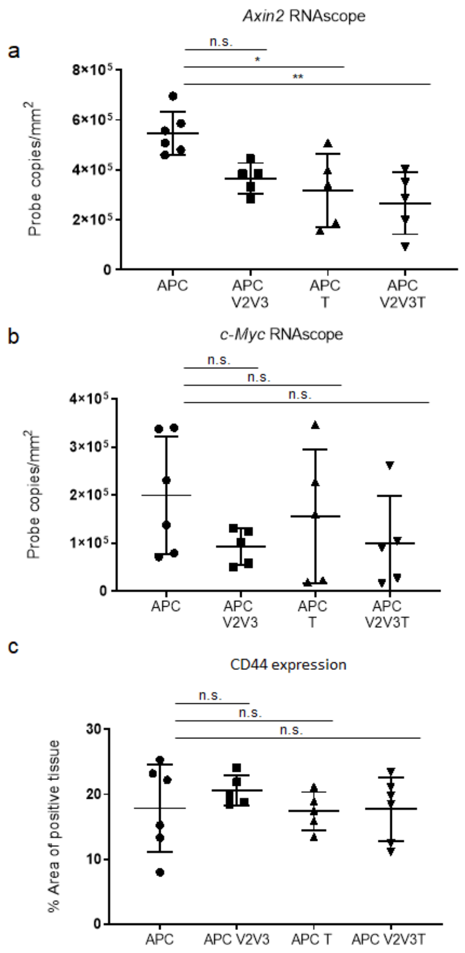

Supplementary Figure 9. WNT target genes are affected following loss of VAV2, VAV3 and TIAM1.

A) Quantification of *Axin2* RNAscope expression. *Vil-CreER*^{T2} *Apc*$^{fl/fl}$ (APC) (n=6 biologically independent animals) vs *Vil-CreER*^{T2} *Apc*$^{fl/fl}$, *Vav2*$^{-/-}$, *Vav3*$^{-/-}$ (APC V2V3) (n=5 biologically independent animals) p = 0.0663. APC vs *Vil-CreER*^{T2} *Apc*$^{fl/fl}$, *Tiam1*$^{-/-}$ (APC T) (n=5 biologically independent animals) *p = 0.0371. APC vs *Vil-CreER*^{T2} *Apc*$^{fl/fl}$, *Vav2*$^{-/-}$, *Vav3*$^{-/-}$ *Tiam1*$^{-/-}$ (APC V2V3T) (n=5 biologically independent animals) (under the control of *Vil-CreER*^{T2}). ** p=0.0072 as determined by a two-tailed Kruskal-Wallis statistical analysis with Dunn's multiple comparisons test. Data are presented as mean +/- SD.

B) Quantification of *c-Myc* RNAscope expression. *Vil-CreER*^{T2} *Apc*$^{fl/fl}$ (APC) (n=6 biologically independent animals) vs *Vil-CreER*^{T2} *Apc*$^{fl/fl}$, *Vav2*$^{-/-}$, *Vav3*$^{-/-}$ (APC V2V3) (n=5 biologically independent animals) p=0.5586. APC vs *Vil-CreER*^{T2} *Apc*$^{fl/fl}$, *Tiam1*$^{-/-}$ (APC T) (n=5 biologically independent animals) p>0.9999. APC vs *Vil-CreER*^{T2} *Apc*$^{fl/fl}$, *Vav2*$^{-/-}$, *Vav3*$^{-/-}$ *Tiam1*$^{-/-}$ (APC V2V3T) (n=5 biologically independent animals) (under the control of *Vil-CreER*^{T2}). p=0.4153 as determined by a two-tailed Kruskal-Wallis statistical analysis with Dunn's multiple comparisons test. Data are presented as mean +/- SD.

C) Quantification of CD44 expression. *Vil-CreER*^{T2} *Apc*$^{fl/fl}$ (APC) (n=6 biologically independent animals) vs *Vil-CreER*^{T2} *Apc*$^{fl/fl}$, *Vav2*$^{-/-}$, *Vav3*$^{-/-}$ (APC V2V3) (N=5 biologically independent animals), APC vs *Vil-CreER*^{T2} *Apc*$^{fl/fl}$, *Tiam1*$^{-/-}$ (APC T) (n=5 biologically independent animals), APC vs *Vil-CreER*^{T2} *Apc*$^{fl/fl}$, *Vav2*$^{-/-}$, *Vav3*$^{-/-}$ *Tiam1*$^{-/-}$ (APC V2V3T) (n=5 biologically independent animals) (under the control of *Vil-CreER*^{T2}). p>0.9999 for each comparison as determined by a two-tailed Kruskal-Wallis statistical analysis test. Data are presented as mean +/- SD.

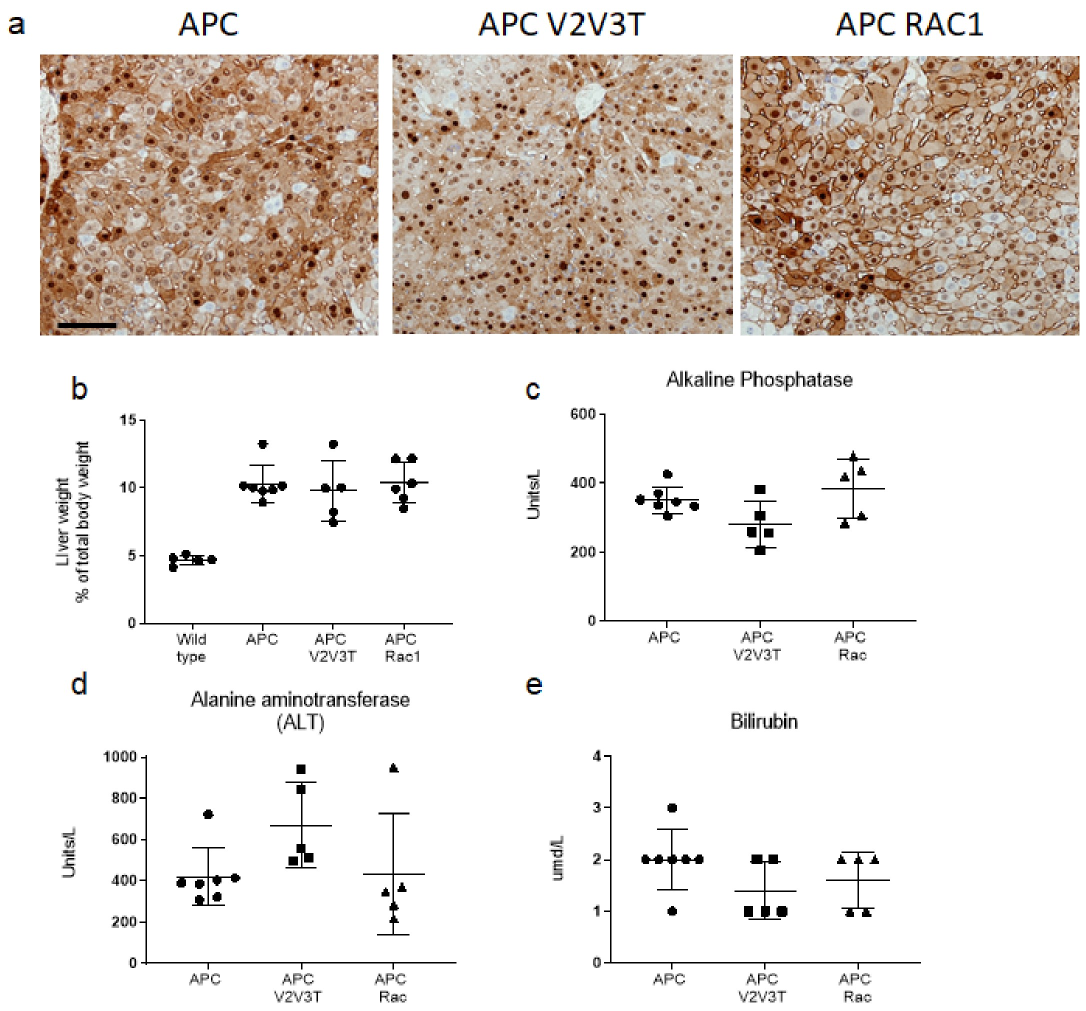

Supplementary Figure 10. The ability to rescue the WNT activation phenotype is intestine specific.

A) β-catenin staining showing nuclear localisation in hepatocytes of all genotypes. Scale bar represents 100 µm.

B) Weight of livers at dissection. Wild-type (WT) (n=5 biologically independent animals). WT vs . *Vil-CreER*^{T2} *Apc*$^{fl/fl}$ (APC) **p=0.0067, APC (n=6 biologically independent animals) vs *Vil-CreER*^{T2} *Apc*$^{fl/fl}$, *Vav2*$^{-/-}$, *Vav3*$^{-/-}$ *Tiam1*$^{-/-}$ (APC V2V3T) (n=5 biologically independent animals) p =0.9071. APC vs *Vil-CreER*^{T2} *Apc*$^{fl/f}$, *Rac1*$^{fl/fl}$ (APC RAC1) (n=6 biologically independent animals) p > 0.9999 as determined by a two-tailed Kruskal-Wallis statistical analysis with Dunn's multiple comparisons test test (under the control of AAV-Cre). Data are presented as mean +/- SD.

C) Biochemical analysis of Alkaline phosphatase n ≥3. *Vil-CreER*^{T2} *Apc*$^{fl/fl}$ (APC) (n=6 biologically independent animals) vs *Vil-CreER*^{T2} *Apc*$^{fl/fl}$, *Vav2*$^{-/-}$, *Vav3*$^{-/-}$ *Tiam1*$^{-/-}$ (APC V2V3T) (n=5 biologically independent animals) p=0.3878. APC vs *Vil-CreER*^{T2} *Apc*$^{fl/f}$, *Rac1*$^{fl/fl}$ (APC RAC1) (n=6 biologically independent animals) p >0.9999 as determined by a two-tailed Kruskal-Wallis statistical analysis with Dunn's multiple comparisons test test (under the control of AAV-Cre). Data are presented as mean +/- SD.

D) Biochemical analysis of ALT. *Vil-CreER*^{T2} *Apc*$^{fl/fl}$ (APC) (n=6 biologically independent animals) vs *Vil-CreER*^{T2} *Apc*$^{fl/fl}$, *Vav2*$^{-/-}$, *Vav3*$^{-/-}$ *Tiam1*$^{-/-}$ (APC V2V3T) (n=5 biologically independent animals) p=0.1763. APC vs *Vil-CreER*^{T2} *Apc*$^{fl/f}$, *Rac1*$^{fl/fl}$ (APC RAC1) (n=5 biologically

independent animals) p > 0.9999 as determined by a two-tailed Kruskal-Wallis statistical analysis with Dunn's multiple comparisons test test (under the control of AAV-Cre). Data are presented as mean +/- SD.

E) Biochemical of analysis of Bilirubin. *Vil-CreER*^{T2} *Apc*$^{fl/fl}$ (APC) (n=6 biologically independent animals) vs *Vil-CreER*^{T2} *Apc*$^{fl/fl}$*, Vav2*$^{-/-}$*, Vav3*$^{-/-}$ *Tiam1*$^{-/-}$ (APC V2V3T) (n=5 biologically independent animals) p=0.4458. APC vs *Vil-CreER*^{T2} *Apc*$^{fl/f}$*, Rac1*$^{fl/fl}$ (APC RAC1) (n=5 biologically independent animals) p >0.9999 as determined by a two-tailed Kruskal-Wallis statistical analysis with Dunn's multiple comparisons test test (under the control of AAV-Cre). Data are presented as mean +/- SD.

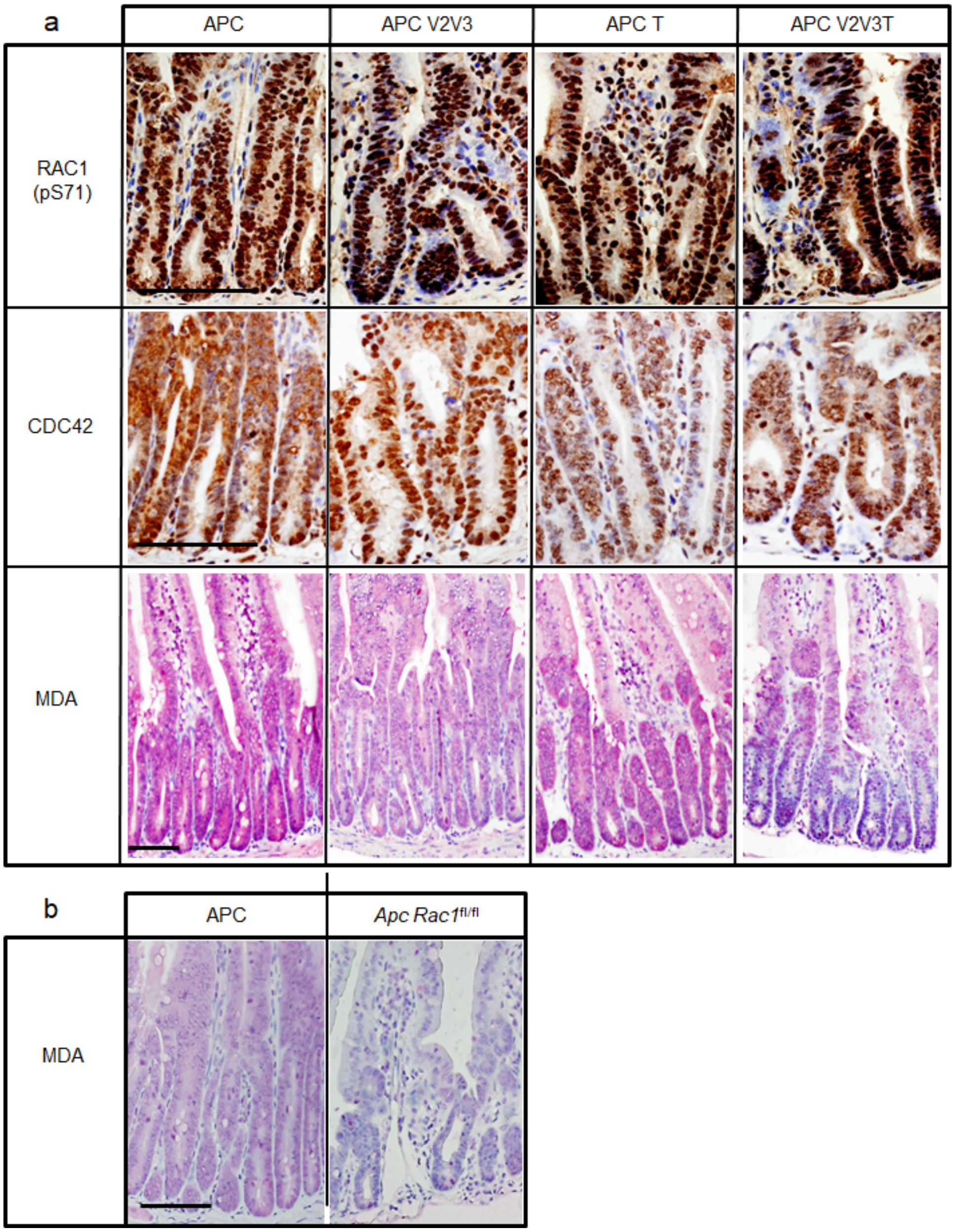

Supplementary Figure 11. Total levels of RAC1 and CDC42 are unaffected whilst Ros is downregulated following loss of the GEFs or RAC1.

A) Immunohistochemical analysis of total RAC1 (pS71), Total CDC42 and MDA in intestines from *Vil-CreER*^{T2} *Apc*$^{fl/fl}$ (APC), *Vil-CreER*^{T2} *Apc*$^{fl/fl}$ *Vav2*$^{-/-}$ *Vav3*$^{-/-}$(APC V2V3) *Vil-CreER*^{T2} *Apc*$^{fl/fl}$ *Tiam1*$^{-/}$ (APC T) and *Vil-CreER*^{T2} *Apc*$^{fl/fl}$ *Vav2*$^{-/-}$ *Vav3*$^{-/-}$ *Tiam1*$^{-/-}$ (APC V2V3T)$^{-}$. Scale bar represents 100 µm.

B) Immunohistochemical analysis of MDA in intestines from *Vil-CreER*^{T2} *Apc*$^{fl/fl}$ (APC) and *Vil-CreER*^{T2} *Apc*$^{fl/fl}$*Rac1*$^{fl/fl}$ (APC *Rac1*$^{fl/fl}$). Scale bar represents 100 µm.

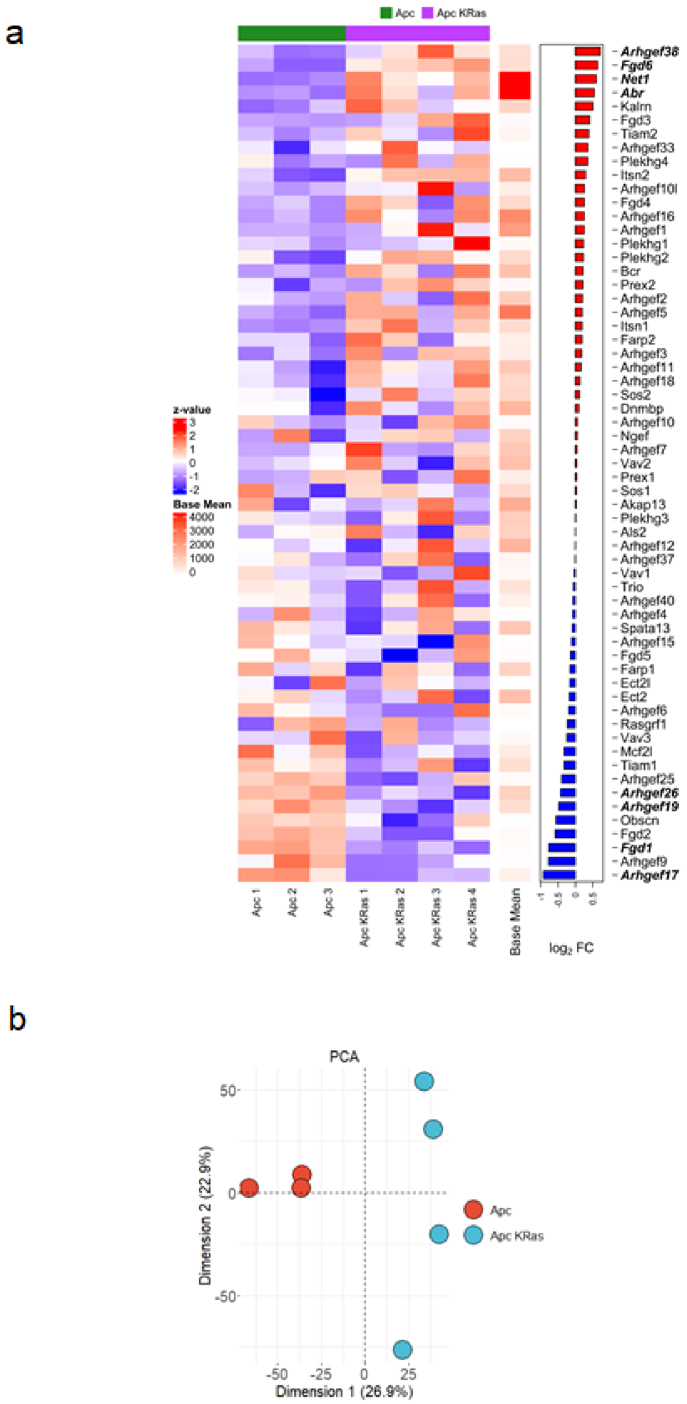

Supplementary Figure 12. RNAseq changes following mutation of oncogenic KRAS.

A) Heatmap derived from RNA-seq analysis comparing whole tissue from *Vil-CreER*^{T2} *Apc*$^{fl/fl}$ (APC) and *Vil-CreER*^{T2} *APC*$^{fl/fl}$ *KRas*$^{G12D/+}$ (APC KRas) intestines. Log$_2$FC of GEF expression displayed on the right, genes significantly deregulated (FDR < 0.05) displayed in bold.

B) PCA of the RNAseq dataset
