# Supplementary material for: A RAC-GEF network critical for early intestinal tumourigenesis
Source: Nat Commun. 2021 Jan 4;12:56. doi: 10.1038/s41467-020-20255-4 (PMC7782582; doi:10.1038/s41467-020-20255-4)
Supplement: Supplementary file 2 — Reporting Summary [file 41467_2020_20255_MOESM2_ESM.pdf]

nature research

Corresponding author(s): Kevin Myant
Andrew Campbell
Owen Sansom

Last updated by author(s): Oct 23, 2020

Reporting Summary

Nature Research wishes to improve the reproducibility of the work that we publish. This form provides structure for consistency and transparency in reporting. For further information on Nature Research policies, see our Editorial Policies and the Editorial Policy Checklist.

Statistics

For all statistical analyses, confirm that the following items are present in the figure legend, table legend, main text, or Methods section.

n/a	Confirmed	
☐	☒	The exact sample size (*n*) for each experimental group/condition, given as a discrete number and unit of measurement
☐	☒	A statement on whether measurements were taken from distinct samples or whether the same sample was measured repeatedly
☐	☒	The statistical test(s) used AND whether they are one- or two-sided<br>*Only common tests should be described solely by name; describe more complex techniques in the Methods section.*
☐	☒	A description of all covariates tested
☐	☒	A description of any assumptions or corrections, such as tests of normality and adjustment for multiple comparisons
☐	☒	A full description of the statistical parameters including central tendency (e.g. means) or other basic estimates (e.g. regression coefficient) AND variation (e.g. standard deviation) or associated estimates of uncertainty (e.g. confidence intervals)
☐	☒	For null hypothesis testing, the test statistic (e.g. F, t, r) with confidence intervals, effect sizes, degrees of freedom and P value noted<br>*Give P values as exact values whenever suitable.*
☒	☐	For Bayesian analysis, information on the choice of priors and Markov chain Monte Carlo settings
☒	☐	For hierarchical and complex designs, identification of the appropriate level for tests and full reporting of outcomes
☒	☐	Estimates of effect sizes (e.g. Cohen's d, Pearson's r), indicating how they were calculated

Our web collection on statistics for biologists contains articles on many of the points above.

Software and code

Policy information about availability of computer code

Data collection	No software was used
Data analysis	FastQ algorithm version 0.11.8<br>Trim Galore version 0.6.4<br>FeatureCounts version 1.6.4<br>GraphPad Prism V7.02<br>HISAT2 version 2.1.0<br>R package DESeq2 version 1.22.2<br>G* power software package 3.1.9.4<br>Halo 3.1.1076.363<br>Limma R<br>Imspector Pro_7_0_153

For manuscripts utilizing custom algorithms or software that are central to the research but not yet described in published literature, software must be made available to editors and reviewers. We strongly encourage code deposition in a community repository (e.g. GitHub). See the Nature Research guidelines for submitting code & software for further information.

Data

Policy information about availability of data

All manuscripts must include a data availability statement. This statement should provide the following information, where applicable:

- Accession codes, unique identifiers, or web links for publicly available datasets
- A list of figures that have associated raw data
- A description of any restrictions on data availability

RNA seq analysis and Fastq files are deposited at Gene Expression Omnibus (NCBI), study accession numbers; GSE112226 and GSE152388. These are both publicly available .
Figure 2, Extended data figure 3, extended data figure 12.

Field-specific reporting

Please select the one below that is the best fit for your research. If you are not sure, read the appropriate sections before making your selection.

☒ Life sciences ☐ Behavioural & social sciences ☐ Ecological, evolutionary & environmental sciences

For a reference copy of the document with all sections, see nature.com/documents/nr-reporting-summary-flat.pdf

Life sciences study design

All studies must disclose on these points even when the disclosure is negative.

Sample size	Power analyses were carried out to determine cohort sizes based on effect size and SD in unpublished experiments previously carried out within the lab using control cohorts of mice and in pilot studies within experimental and control cohorts. Power analyses were carried out using the G* power software package 3.1.9.4 (HHU Dusseldorf).
Data exclusions	NA - no animals or data were excluded
Replication	For all in vivo analysis each data point represents a biologically unique animal. The clonogenecity experiment was comprised of 3 biologically distinct replicates and at least 3 technical replicates.
Randomization	There were no treatment studies except for the DSS study in which all animals in the study received the same treatment. To minimise genetic variability, all experimental and control animals were generated from individual breeding colonies. Control and experimental animals were co-housed independent of genotype and cohorts were comprised of a balance of both male and female animals.
Blinding	Data collection and analysis were carried out in a blinded manner and researchers were unblinded post-analysis.

Reporting for specific materials, systems and methods

We require information from authors about some types of materials, experimental systems and methods used in many studies. Here, indicate whether each material, system or method listed is relevant to your study. If you are not sure if a list item applies to your research, read the appropriate section before selecting a response.

Materials & experimental systems

n/a	Involved in the study	
☐	☒	Antibodies
☒	☐	Eukaryotic cell lines
☒	☐	Palaeontology and archaeology
☐	☒	Animals and other organisms
☒	☐	Human research participants
☒	☐	Clinical data
☒	☐	Dual use research of concern

Methods

n/a	Involved in the study	
☒	☐	ChIP-seq
☒	☐	Flow cytometry
☒	☐	MRI-based neuroimaging

Antibodies

Antibodies used	Primary antibodies and concentrations were used as follows; VAV1 (1:50; Cell Signalling #2502), RAC1 (1:500; Abcam Ab203884); CDC42 (1:200; Abcam Ab155940) (66); Active-RAC1 (1:500, NewEast Biosciences; cat# 26903) (67), Cleaved-caspase 3 (1:500; Cell Signalling; cat# 9661) (68), E-Cadherin (clone 24E10, 1:200; Cell Signalling cat#3195) (69), SOX9 (1:500; Millipore; cat# AB5535), β-catenin (1:50; BD Biosciences; cat#610154) (70), CD44 (1:100; BD Biosciences; cat# 550538) (71), MDA (1:100; Abcam Ab6463) (13); VAV2 (1:200; Abcam Ab79182); VAV3 (1:200; Abcam; Ab203315); CTGF (1:200; Abcam; Ab6992) (48). Secondary HRP-tagged

	secondaries were used as follows; Dako Envision+ system HRP goat anti-mouse (neat; Dako K4001), Dako Envision+ system HRP goat anti-rabbit (neat; Dako K4003). Secondary fluorescent antibodies were used as follows; Alexafluor-488 (1:200; ThermoFisher; cat # A11034), AlexaFluor 594 (1:200; ThermoFisher; cat #A11032). Proliferation was determined by quantifying BrdU incorporation (1:500; BD Biosciences Cat # 347580). primary anti-Rac1 monoclonal antibody for the Rac1 pull downs(Cytoskeletion; #ARC03; 1:500) References provided in manuscript
Validation	Vav1 - Validated in house by IHC using whole body knockout intestinal mouse tissue.<br>Rac1 - Validated in house by IHC using intestinal tissue from a Vil-CreERT2, Rac1fl/fl conditional knockout mouse post induction. Here we observed no staining in the intestinal epithelium (extended data 2b).<br>CDC42 - validated by the manufacturer (abcam) using positive control cells including; A431, C6, Hep2 and Hela cells in addition to FFPE human pancreatic tissue. This antibody was validated to work in wester blot, immunoflourescence and IHC-P<br>Active Rac1 - validated by the manufacturer (new east biosciences) using GTP loaded MEFs for both pull downs and IF<br>Cleaved caspase 3 - Validated by the manufactrer (CST) using staurosporin treated HeLa, NIH/3T3 and C6 cells for western blot and immunofluorescence. Use for IHC was validated by embedded Jurkat cells untreated (negative control) or etoposide-treated (positive control).<br>E-Cadherin - validated by Bai et al 2015 where they showed deletion of Yap results in the disruption of E-cadherin junctions.<br>Sox9 - validated by Vng et.al 2015 using tissue from a Sox9 conditional knockout mouse<br>B-catenin - antibody was generated and validated by Ozawa at al 1990.<br>CD44 - validated by the manufacturer (BD biosciences) during development. Shown to work in IHC-P using mouse spleen.<br>MDA - used as a measure of ROS damage by Cheung et al. 2015<br>Vav2 - Validated by iHC in extended data 4<br>Vav3 - Validated by IHC in extended data 4<br>CTGF - Validated in Diamtopolou et al. 2017<br>primary anti-Rac1 monoclonal antibody for the Rac1 pull downs (Cytoskeletion; #ARC03; 1:500) - Validated by manufacturer by western blot of purified platelet extracts.

Animals and other organisms

Policy information about studies involving animals; ARRIVE guidelines recommended for reporting animal research

Laboratory animals	Page 14. Mice of both sexes on a mixed-strain background were used. Mice were induced at between 6 and 10 weeks of age and 20-25g. Mice were housed in conventional caging, with environmental enrichment on a 12 hour light-dark cycle in a temperature and humidity controlled environment, with access to food and water ad libitum. Mice were assessed for symptoms of ill health at least 3 times per week.
Wild animals	No wild animals were used in this study.
Field-collected samples	No field collected samples were used in this study.
Ethics oversight	Experiments were performed under UK Home Office license approved by the University of Glasgow Animal Welfare Ethical Review Board (H/O license Owen Sansom PPL 70-8646)

Note that full information on the approval of the study protocol must also be provided in the manuscript.
